# Characterizing the Fate of Anti‐CS1 Nanobody Displaying Extracellular Vesicles in Multiple Myeloma

**DOI:** 10.1002/jev2.70325

**Published:** 2026-06-17

**Authors:** Michiel De Coster, Lukas Hyka, Sophie De Cock, Sofie Meeussen, Zuowei Wang, Chenggong Tu, Sophie Hernot, Nick Devoogdt, Guido David, Kim De Veirman, Karin Vanderkerken, Eline Menu, Pascale Zimmermann, Elke De Bruyne

**Affiliations:** ^1^ Translational Oncology Research Center (TORC), Team Hematology and Immunology (HEIM) Vrije Universiteit Brussel Brussels Belgium; ^2^ Laboratory for Extracellular Vesicle Research, Department of Human Genetics Katholieke Universiteit Leuven Leuven Belgium; ^3^ Molecular Imaging and Therapy Research Group (MITH) Vrije Universiteit Brussel Brussels Belgium; ^4^ Équipe Labellisée Ligue 2018, INSERM 1068, CNRS 7258, Institut Paoli Calmettes, Centre de Recherche en Cancérologie de Marseille Aix Marseille Université Marseille France

**Keywords:** anti‐CS1 nanobodies, biodistribution, extracellular vesicles, multiple myeloma, targeted delivery

## Abstract

Extracellular vesicles (EVs) are promising delivery vehicles capable of transporting therapeutic agents across biological barriers. However, native EVs primarily accumulate in liver, spleen and lungs, limiting targeted delivery to disease sites. To enhance their targeting efficiency for the plasma cell cancer multiple myeloma (MM), localized in the bone marrow (BM), we engineered HEK293‐derived EVs to display a nanobody (Nb) against the MM cell surface marker CS1. We confirmed enrichment of the Nb construct on engineered EVs and binding of α‐CS1 EVs to CS1. *In vitro*, we found enhanced α‐CS1 EV uptake by MM cell cultures. *In vivo*, we first compared the biodistribution of HEK293‐derived native EVs in healthy and MM‐bearing mice. Although native EVs reached the BM in both groups, MM‐bearing mice showed increased liver and lung accumulation together with reduced BM delivery. α‐mCS1 EV delivery to the BM of MM‐bearing mice was only slightly increased compared to native EVs, while off‐target accumulation also increased. At the cellular level, no changes in EV delivery to MM cells were detected. In conclusion, while CS1 targeting enhances *in vitro* EV uptake by MM cells, *in vivo* biodistribution remains suboptimal. Further optimization is needed to improve EV‐based drug delivery for MM.

## 1 Introduction

Multiple myeloma (MM) is an incurable plasma cell cancer that mainly resides in the bone marrow (BM). First line treatment comes as a triple or quadruple therapy, consisting of proteasome inhibitors (PIs; bortezomib and carfilzomib) and immunomodulatory drugs (IMiDs; lenalidomide and pomalidomide), together with corticosteroids and monoclonal antibodies targeting CD38 (daratumumab or isatuximab) (Callander et al. 2024). If patients are eligible for transplant, an autologous stem cell transplantation is often performed afterwards. However, MM is still incurable due to recurring relapses, either due to the development of progressive drug resistance or the necessity to discontinue treatment due to toxicity from prolonged therapy. Although novel immunotherapies like B‐cell maturation antigen (BCMA)‐targeted CAR T cell therapies (Roex et al. 2020) and bispecific T cell engagers (Ravi and Costa 2023) have shown promising results in relapsed/refractory MM, they are also associated with neurotoxicity, cytokine release syndrome and depletion of non‐malignant cells (Cappell and Kochenderfer 2023). Additionally, the overall MM incidence and mortality continue to rise (Cowan et al. 2016), underscoring the critical need for innovative therapeutic strategies that can effectively target MM cells while minimizing off‐target effects and overcoming drug resistance. To address these limitations, we and others (Yuan et al. 2024) propose utilizing targeted extracellular vesicles (EVs) for drug delivery to MM cells, aiming to enhance treatment efficacy and reduce toxicities associated with long‐term therapy.

EVs are nanosized biological particles containing proteins, lipids, nucleic acids and metabolites enclosed by a bilipid membrane. EVs encompass a heterogeneous population, including smaller exosomes of endosomal origin and larger microvesicles shed from the cell membrane, among other subtypes (Couch et al. 2021; Wakker et al. 2023). In this study, we utilized the general term ‘EVs’ throughout, acknowledging the inherent mixture of large (lEVs) and small EVs (sEVs) within our preparations (Théry et al. 2018). EVs can be leveraged for therapeutic purposes by engineering them to carry customized cargo within their lumen and display specific molecules on their surface (Yang et al. 2024). Furthermore, EVs can be loaded with existing therapeutics, increasing the therapeutic index of the encapsulated drugs due to less renal clearance, increased stability, improved site‐specific delivery, enhanced cellular uptake and reduced drug resistance (Kim et al. 2024). These properties have led them to emerge as promising drug delivery systems, with potential applications in a wide range of diseases (Ghodasara et al. 2023), including cancer. For example, the phase I clinical trial iEXPLORE currently explores the use of mesenchymal stromal cell (MSC)‐derived EVs loaded with siRNA targeting KRAS^G12D^ in pancreatic cancer (Kamerkar et al. 2017; Mendt et al. 2018) (NCT03608631). First results from this study are reassuring and encouraging, with no dose‐limiting toxicities observed, no maximum tolerated dose reached and evidence of early biological signals, and final results are expected in 2027 (Kalluri et al. 2025). Furthermore, the surface of EVs can be engineered to exert therapeutic effects (Dooley et al. 2021) or alter EV biodistribution and target cell specificity (Alvarez‐Erviti et al. 2011; Hyka et al. 2026; Pham et al. 2021).

In the context of MM, bortezomib‐loaded monocyte‐derived EVs targeting BCMA (anti‐BCMA EVs) have shown potent anti‐myeloma effects in an orthotopic murine model (Yuan et al. 2024). However, challenges, such as prevalent antigen escape upon treatment pressure for CD38 (Portuguese et al. 2023) and BCMA (Firestone et al. 2024)‐targeting immunotherapies and off‐target effects, necessitate the exploration of alternative targets to enhance therapeutic efficacy and broaden patient applicability. CS1 (SLAMF7), a glycoprotein present on MM cells, represents such a promising alternative. Therapies directed against CS1 have demonstrated efficacy in clinical settings (van de Donk et al. 2016) and its distinct expression profile (Chu et al. 2023) alongside that of BCMA, makes it an attractive candidate for targeted delivery approaches in MM. Moreover, we previously showed specific retention of our anti (α)‐CS1 Nbs in the MM‐associated organs of the syngeneic immunocompetent 5T33 and 5TGM1 murine models (De Veirman et al. 2021).

In the present study, we investigated the potential of HEK293‐derived EVs as delivery vehicles for the treatment of MM. HEK293 cells were chosen as EV source, as they are easily engineered and capable of producing scalable, reproducible and clinically compliant EVs with minimal immunogenicity (Tan et al. 2021). To increase the targeting of EVs towards MM‐associated tissue and organs, we engineered HEK293‐derived EVs to display α‐CS1 Nbs on their surface and evaluated EV binding to soluble CS1, biodistribution in healthy and myeloma‐bearing mice and cellular uptake in relevant MM models. This approach seeks to advance the field of targeted delivery systems in MM, offering insights into the potential of EV‐based therapies to selectively target malignant plasma cells while sparing healthy tissue.

## 2 Materials and Methods

### 2.1 Cells

Human embryonic kidney 293 (HEK293) cells were cultured in DMEM (Thermo Fisher Scientific, Waltham, MA, USA) supplemented with 10% fetal calf serum (FCS; Hycone, Logan, UT, USA), 100 U/mL penicillin/streptomycin and 2 mM L‐glutamine (Thermo Fisher Scientific). The human myeloma cell line (HMCL) OPM‐2 was cultured in RPMI 1640 medium (Thermo Fisher Scientific) supplemented with 10% FCS and 2 mM L‐glutamine. All cells were cultured in a humidified atmosphere at 37°C and 5% CO_2_, regularly tested for *Mycoplasma* contamination and authenticated by short‐tandem repeat profiling. Cells were acquired from American Type Culture Collection (ATCC; Molsheim, France).

### 2.2 Mice

Female C57BL/KalwRij mice were acquired from Envigo Laboratories (Horst, The Netherlands). Housing, treatment and experimental designs were approved by the Ethical Committee for Animal Experiments of the Vrije Universiteit Brussel (CEP 23‐281‐8 and CEP 25‐281‐8). Per experiment, all mice were housed on the same rack to minimize confounding factors. For tumour inoculation, mice (aged between 6 and 14 weeks) were intravenously (i.v.) injected with 5 × 10^5^ murine 5T33MM cells. Mice were injected with EVs in a blinded manner, while the analyses were performed non‐blinded. To isolate the cells from the MM infiltrated organs of end‐stage diseased mice, the BM was flushed from the legs while the spleen and spine were crushed. Obtained cell suspensions were filtered through a 70 µm nylon filter and incubated with red blood cell lysis buffer (160 mM NH_4_Cl, 170 mM Tris, pH 7.2) for 2 mins to remove the red blood cells. Next, the cells were washed and resuspended in RPMI 1640 medium + 10% FCS. All experiments were performed in accordance with relevant guidelines and regulations, taking the ARRIVE Essential 10 guidelines in account.

### 2.3 Molecular Cloning

DNA sequences encoding α‐hCS1 (VHH‐6) (Hanssens et al. 2024), α‐mCS1 (sdAb1) (De Veirman et al. 2021) and control (R3B23) Nbs (De Veirman et al. 2021; Hanssens et al. 2024; Lemaire et al. 2014) fused to SDC1CTF via the juxtamembrane domain of CD4 (CD4JM) were synthesized as gBlocks (Integrated DNA Technologies (IDT), Coralville, IA, USA), with the coding sequences flanked by *attB* sites. Of note, we previously showed that both VHH‐6 and sdAb1 strongly bind to cell‐expressed CS1 protein on CS1 positive MM cells *in vitro* and efficiently target MM lesions *in vivo*. Moreover, for VHH‐6 we also showed that CAR T cells employing VHH‐6 for antigen recognition showed binding to CS1 positive OPM‐2 cells and subsequent CAR T cell activation (De Veirman et al. 2021; Hanssens et al. 2024). Following a BP reaction (Thermo Fisher Scientific) in the pDONR Zeo entry vector, competent *E. coli* DH5α were transformed by heat shock at 42°C for 45 s, transferred to ice and cooled for 2 mins. Transformed cells were then incubated with 900 µL Super Optimal broth with Catabolic repression (S.O.C.) medium for 45 mins at 37°C, while shaking at 250 RPM. Next, cells were plated on LB agar plates containing 100 µg/mL zeocin and incubated overnight at 37°C. Colonies were picked, cultured and screened by sequencing.

Nb‐SDC1CTF encoding sequences were transferred from the pDONR Zeo entry vector to a pcDNA3 expression vector containing *attR* sites, allowing an LR reaction (Thermo Fisher Scientific). Competent *E. coli* DH5α were transformed and plated as described above, but now selecting for ampicillin (100 µg/mL). After confirming the correct sequence, the plasmids were amplified and isolated using a maxiprep DNA isolation kit.

The Nb‐CD4JM‐SDC1CTF fusion protein is based on the finding that substituting the JM domain of SDC1 with the JM domain of CD4 confers protease‐resistance to SDC1 (Fitzgerald et al. 2000). However, the sequence that the authors report deviates from the Genome Reference Consortium Human Build 38 (GRCh38) consensus sequence. To avoid confusion, we clearly state that in the present writing the GRCh38 sequence of CD4JM (GenBank ID: QDC22486.1) was used, as shown in Figure 2(A). For the sequences and binding affinities of the Nbs, please refer to Table S1.

### 2.4 Generation of HEK293 Cell Lines Stably Expressing the Nanobody (Nb)‐SDC1CTF Fusion Proteins

The day before transfection, 4 × 10^5^ HEK293 cells were plated in 6‐well plates. Additionally, 2.5 µg of the plasmid DNA was linearized overnight using MfeI‐HF for the empty vector and (myc)‐α‐hCS1 Nb and α‐mCS1 Nb‐CD4JM‐SDC1CTF vector, while PvuI was used for the control Nb‐CD4JM‐SDC1CTF vector according to the manufacturer's instructions. The cut plasmid was purified from any uncut plasmid by agarose gel electrophoresis. Next, the transfection mix, containing 30 µL of DNA and 3 µL of X‐tremeGene 9 (Sigma‐Aldrich) in a total volume of 100 µL diluted with OptiMEM (Gibco), was mixed and incubated for 15 mins at room temperature. Meanwhile, the cells were supplied with fresh growth medium, after which the transfection mix was added dropwise to the cells. Six hours later, the medium of the cells was refreshed with growth medium. Two days after transfection, the cells were plated in 10 cm dishes and further cultured in growth medium supplemented with 300 µg/mL G418.

### 2.5 Preparation of EV‐Depleted Serum

FCS was thawed overnight at 4°C and subsequently heated to 56°C for 30 mins. Next, the serum was filtered through a 0.22 µm filter, transferred to ultracentrifuge tubes (ref.: 326823, Beckman Coulter, Fullerton, CA, USA) and centrifuged at 28,000 RPM using an SW28 rotor for 18 h at 4°C. The collected supernatant was again filtered through a 0.22 µm filter, after which aliquots of EV‐depleted serum (EDS) were stored at −20°C until use.

### 2.6 Western Blot

Cell pellets were lysed with lysis buffer composed of 50 mM Tris, 150 mM NaCl, 1% Nonidet P40 and 0.25% sodium deoxycholate. Additionally, the lysis buffer contained the following protease and phosphatase inhibitors: Na_3_VO_4_ (4 mM), Na_4_P_2_O_7_ (1 mM), aprotinin (2 µg/mL), leupeptin (50 µg/mL), trypsin inhibitor (500 µg/mL), benzamidine (10 µM), PNP benzoate (2.5 mM), NaF (50 mM), ethylenediaminetetraacetic acid (5 mM), 4‐(2‐aminoethyl) benzenesulfonyl fluoride hydrochloride (1 mM) and pepstatin A (50 µg/mL). The lysate was centrifuged for 5 mins at 21,100 g to remove cell debris. The supernatant was stored at −80°C until use.

The protein concentration of cell lysates was determined by the Pierce BCA Protein Assay Kit according to the manufacturer's instructions. Lysates were then diluted 1:1 with loading buffer consisting of a Laemmli sample buffer with β‐mercaptoethanol (1:20). To analyse the proteins isolated from EVs, they were diluted 4:1 with 5X Pierce^TM^ Lane Marker Reducing Sample Buffer instead. After mixing with loading buffer, samples were boiled for 5 mins and loaded on the gel.

Proteins were separated using sodium dodecyl sulphate (SDS) polyacrylamide electrophoresis (SDS‐PAGE) and transferred to a Hybond‐C extra 0.45 µm nitrocellulose or polyvinylidene difluoride (PVDF) membrane. Next, the membranes were blocked with blocking buffer composed of tris‐buffered saline (TBS), 5% (w/v) milk powder and 0.1% Tween 20 for 1 h. The membrane was then incubated overnight with blocking buffer containing the primary antibody. Next, the membrane was washed 3 times with TBS containing 0.1% Tween 20 (TBS‐T) for 5 mins and incubated for at least 1 h with an HRP‐coupled secondary antibody in blocking buffer at room temperature. The membranes were developed by chemiluminescence and were visualized using the Li‐Cor Odyssey Fc. Images were processed using the Image Studio Lite Software and quantifications were made using ImageJ.

Primary and secondary antibodies used can be found in Table S2.

### 2.7 EV Isolation by Ultracentrifugation

For all experiments except for SEC EVs for EV—CS1 bindings assays and concentrated conditioned medium (CCM) EVs for dSTORM super‐resolution microscopy, EVs were isolated by ultracentrifugation as follows: 7 × 10^6^ HEK293 cells (non‐engineered HEK293 cells for the experiment comparing the distribution of native EVs in naïve versus MM‐diseased mice and HEK293 cells stably expressing the Nb constructs for all other experiments) were seeded in T175 flasks. Cells were allowed to attach overnight and the medium was replaced to DMEM + 5% EDS. Two days later, the CM was collected and centrifuged for 5 mins at 500 × g. The supernatant was centrifuged again for 20 mins at 2000 × g, 4°C. When separating small EVs (sEVs) from large EVs (lEVs), the supernatant was first centrifuged at 10,000 × g to pellet lEVs before isolating sEVs. To isolate sEVs, the resulting supernatant was transferred to ultracentrifuge tubes and centrifuged at 113,000 × g, for 1h 30 min at 4°C. The resulting pellet was resuspended in PBS, transferred again to ultracentrifuge tubes (ref. 331372, Beckman Coulter) and centrifuged a second time at 107,000 × g, for 1h 30 min at 4°C. This pellet finally was resuspended in PBS and stored at 4°C when used within 24 h, and at −80°C when used later. EVs were used within 2 months after isolation. The particle concentration of EV samples was determined by Zetaview Nanoparticle Tracking Analysis (NTA) using the following acquisition settings: sensitivity: 70, shutter: 100, frame rate: 30 fps. The presence of common EV markers and absence of calreticulin was validated by western blot (Figure S7).

### 2.8 EV Isolation by Size Exclusion Chromatography

HEK293 cells stably transfected with either the empty vector (mock) or Nb‐SDC1CTF fusion proteins were grown to 90% confluence in DMEM + 5% EDS. Next, the medium was replaced with DMEM containing no serum to avoid clogging of filters and columns by concentrated FCS in downstream procedures. The cells were incubated at 37°C and 5% CO_2_ for two days. Afterwards, the CM was transferred to 50 mL falcons and centrifuged at 500 × g for 5 mins at room temperature. The resulting supernatant was again centrifuged at 2000 × g for 20 mins at 4°C. This final supernatant, being CM clarified from cells and apoptotic bodies, was then stored at −80°C until further use. The day before use, the CM was thawed overnight at 4°C and concentrated to maximum 1 mL using Amicon Ultra‐15, 30 kDa centrifugal filters. The concentrated CM was loaded on qEV1 35 nm size‐exclusion chromatography (SEC) columns (Izon Science, Christchurch, New Zealand). The buffer volume of 4.7 mL was discarded, and the following 2.8 mL of eluate (4 pooled fractions of 700 µL) was collected as EVs. The particle concentration of EV samples was again determined by Zetaview NTA.

### 2.9 dSTORM Super‐Resolution Microscopy

EVs prepared by differential ultracentrifugation (dUC EVs) were stained with AF561‐conjugated anti‐pan‐tetraspanin antibodies (recognizing CD63, CD81 and CD9; ONI) and AF647‐coupled anti‐V_HH_ antibody (Jackson ImmunoResearch, Cambridge, United Kingdom). EVs were immobilized via phosphatidylserine binding (ONI) on a PEGgylated coverslip, prepared as previously described (Margeat et al. 2006) and imaged using the ONI super‐resolution Nanoimager. The power of the lasers was 12% and 80% for the 640 nm and 560 nm wavelengths, respectively. Images were analysed with CODI software (ONI). Signal from at least 4 localizations in 561 channel (tetraspanins) was classified as a single EV. Signal of 4 localizations in 561 channel and 2 localizations in 647 channel (Nb) was taken as a measurement of single Nb‐displaying EV.

The CCM EVs were prepared by incubating the HEK293 cells in serum‐free medium 16 h prior to the EV collection, removal of 10K pellet by centrifugation and concentration of the supernatant by 10 kDa Amicon filters (Sigma‐Aldrich). Next, the prepared CCM EVs were stained and analysed as described above.

### 2.10 EV Labelling With DiO or DiR

EVs were incubated with 5 µM DiO or DiR for 30 mins at 37°C, protected from light. Next, the dyed EVs were washed in 12 mL PBS and centrifuged at 107,000 x g using the SW41Ti rotor for 1h 30 min at 4°C. Obtained pellets were resuspended in PBS in case of *in vivo* studies or in serum‐free RPMI 1640 medium for *in vitro* experiments. For indicated experiments, dye controls consisted of 5% EDS control medium (DMEM + 5% exosome‐depleted FCS + supplements), incubated in a cell‐free flask and processed identical to the CM.

### 2.11 EV—CS1 Binding Assays

EVs were isolated by SEC, and 1 × 10^10^ particles/mL were incubated with 770 ng/mL human (R&D Systems) or murine CS1 (U‐Protein Express, Utrecht, The Netherlands) for 1 h at 4°C. Next, the samples were re‐isolated using qEV1 35 nm SEC columns to allow separation of the EV‐bound CS1 and free soluble CS1 protein. After discarding the buffer volume, 3 fractions of 3.5 mL were collected. These fractions were concentrated to 40 µL using Amicon 0.5 mL 30 kDa Ultra Centrifugal Filters and stored at −20°C until western blot analysis.

### 2.12 EV—Cell Uptake Assays

Nine billion EVs isolated by ultracentrifugation were labelled with DiO as described above and finally resuspended in 110 µL. To assess the uptake of the α‐hCS1 Nb displaying EVs by human CS1^+^ MM cells, 100 µL of the EVs was added to 5 × 10^4^ OPM‐2 cells in flat‐bottom 96‐well plates, in a total volume of 200 µL. Two and four hours after incubation at 37°C, 100 µL of the cell suspension was transferred to flow cytometry tubes and washed once using PBS. Next, the cells were resuspended in 300 µL FACSFlow (BD Biosciences, Aalst, Belgium) and the % DiO^+^ cells and MFIs were determined using the BD Accuri C6 Flow Cytometer.

To assess the uptake and specificity of the α‐mCS1 Nb displaying EVs towards murine CS1^+^ MM cells, 20 µL of DiO‐labelled EVs (prepared as described above) was added to 1 × 10^5^ mononuclear cells isolated from the BM and spleen of 5T33MM bearing mice in flat‐bottom 96‐well plates. After 4 h of incubation at 37°C, all cells were transferred to flow cytometry tubes and washed with FACS (PBS + 0.5% FCS + 2 mM ethylenediaminetetraacetic acid (EDTA)) buffer. Next, the cells were stained with 1/100 PE‐coupled anti‐mouse CS1 (Biolegend, San Diego, CA, USA, Catalog# 152006) in 100 µL FACS buffer for 30 mins at 4°C. After washing with FACS buffer, the cells were fixed and permeabilized using Cytofix/Cytoperm (BD Biosciences) for 12 mins at 4°C. Afterwards, the cells were washed again and stained with AF647‐coupled anti‐idiotype 3H2 (Vanderkerken et al. 1997) (1/650) in 100 µL Cytowash/Cytoperm (BD Biosciences). The cells were then washed again twice with FACS buffer and finally resuspended in 300 µL FACS buffer. The % DiO^+^ cells, within CS1^+/−^ and MM^+/−^ subpopulation, and the MFIs were determined using the BD FACSymphony A1 cell analyser.

### 2.13 EV Biodistribution in Mice

To compare the biodistribution of native EVs between naïve and MM‐bearing mice, as well as α‐mCS1 EVs versus native and control Nb EVs in 5T33MM mice, EVs were harvested from a total of 15 T175 flasks per mouse. EVs were produced in three separate batches, with the different conditions processed in parallel and the resulting EVs isolated via ultracentrifugation as described above. Isolated EVs were stored at −80°C. On the day of injection, EVs were thawed and conditions were normalized to the same particle concentration (ranging from 2.8 x 10^10^–1.1 x 10^11^ between experiments) in 3 mL PBS, after which EVs were dyed as described above. An aliquot of 15 µL was used for Zetaview NTA and WB quality assessments.

To perform dose‐escalations of α‐mCS1 Nb EVs in 5T33MM mice, the respective engineered HEK293 cells (35 × 10^6^) were seeded in an 8‐layer CELLdisc (Greiner Bio‐One, Kremsmünster, Austria) to generate sufficient amounts of EVs. Three days later, the culture medium was replaced every two days, alternating between DMEM + 5% EDS and medium without serum, for a period of three weeks. Collected CM was centrifuged at 2000 x g for 20 mins at 4°C, after which the supernatant was concentrated using 100 kDa Amicon Ultra‐15 filters. All concentrated EV collections were then stored at −80°C. On the day of the experiment, all EV samples were pooled and the EVs were subsequently isolated and DiR‐labelled as before. After labelling, the dyed EVs were washed in 6 × 12 mL PBS and centrifuged at 107,000 x g using the SW41Ti rotor for 1h 30mins at 4°C. The dyed EVs were resuspended in 1.1 mL PBS and the particle number was determined by Zetaview NTA. EVs were then diluted ½ and ¼ and injected in a final volume of 200 µL per mouse. An aliquot of 10 µL was used for Zetaview NTA and WB quality assessments. The undiluted, ½ diluted and ¼ diluted injections contained respectively 8 × 10^10^, 4 × 10^10^ and 2 × 10^10^ EVs per mouse.

Eighteen days after 5T33 tumour inoculation, all mice (including the naïve mice, where relevant) were injected simultaneously via the tail vein with 200 µL of DiR labelled EVs. Twenty‐four hours post‐injection, the mice were sacrificed and skinned. Using the Fluobeam800 camera (Fluoptics, Grenoble, France), an image was taken of the front and the back of the mice. After whole body imaging, the liver, spleen, lungs, heart, kidneys, legs and spine were isolated. To evaluate the biodistribution in the individual mice, all extracted organs of each mouse were compiled in one frame and imaged. To appreciate differences of EV uptake between mice, each type of organ from all mice was compiled in a single frame and imaged. All images were taken with 10, 30, 50, 100, 200, 300, 500 and 1000 ms exposure settings. For the analyses, the longest exposure time without any saturation of the signal was used. In case of no saturation, 1000 ms exposure was used. To quantify the DiR signal, the mean grey values were determined using ImageJ.

### 2.14 Determination of EV Specificity Towards MM Cells in 5T33MM Mice

To determine the % DiR^+^ MM and non‐MM cells of mice injected with DiR‐labelled EVs, mononuclear cells isolated from the legs, spine and spleen (as described above) were first washed with 500 µL PBS. Cells were then stained with Fixable Viability Dye eFluor^TM^ 506 (1:1000) (Thermo Fisher Scientific) at 4°C. To stain CS1^+^ cells, samples were first incubated with F_c_R Blocking Reagent (1:200) (Miltenyi Biotec, Bergisch Gladbach, Germany) for 10 mins at 4°C, and then stained using PE anti‐mCS1 (1:100) (Biolegend, catalog# 152006). Next, cells were fixed and permeabilized by incubation with 100 µL BD Cytofix/Cytoperm for 17 mins at 4°C. Afterwards, the cells were washed twice and stained with AF647‐coupled anti‐idiotype 3H2 (Vanderkerken et al. 1997) (1/650) in 100 µL Cytowash/Cytoperm. The cells were then washed again twice with FACS buffer and finally resuspended in 300 µL FACS buffer. Flow cytometric analysis was performed on a BD FACSymphony A1. To be included in our experiments, mice needed to show at least 15% of MM cells in spleen, legs and spine.

### 2.15 Statistical Analysis

Statistical analyses were performed with GraphPad Prism 8.01. All data are represented as mean ± standard deviation (SD).

## 3 Results

### 3.1 The Distribution of Native HEK293 EVs is Altered in Mice With Multiple Myeloma

**FIGURE 1:**
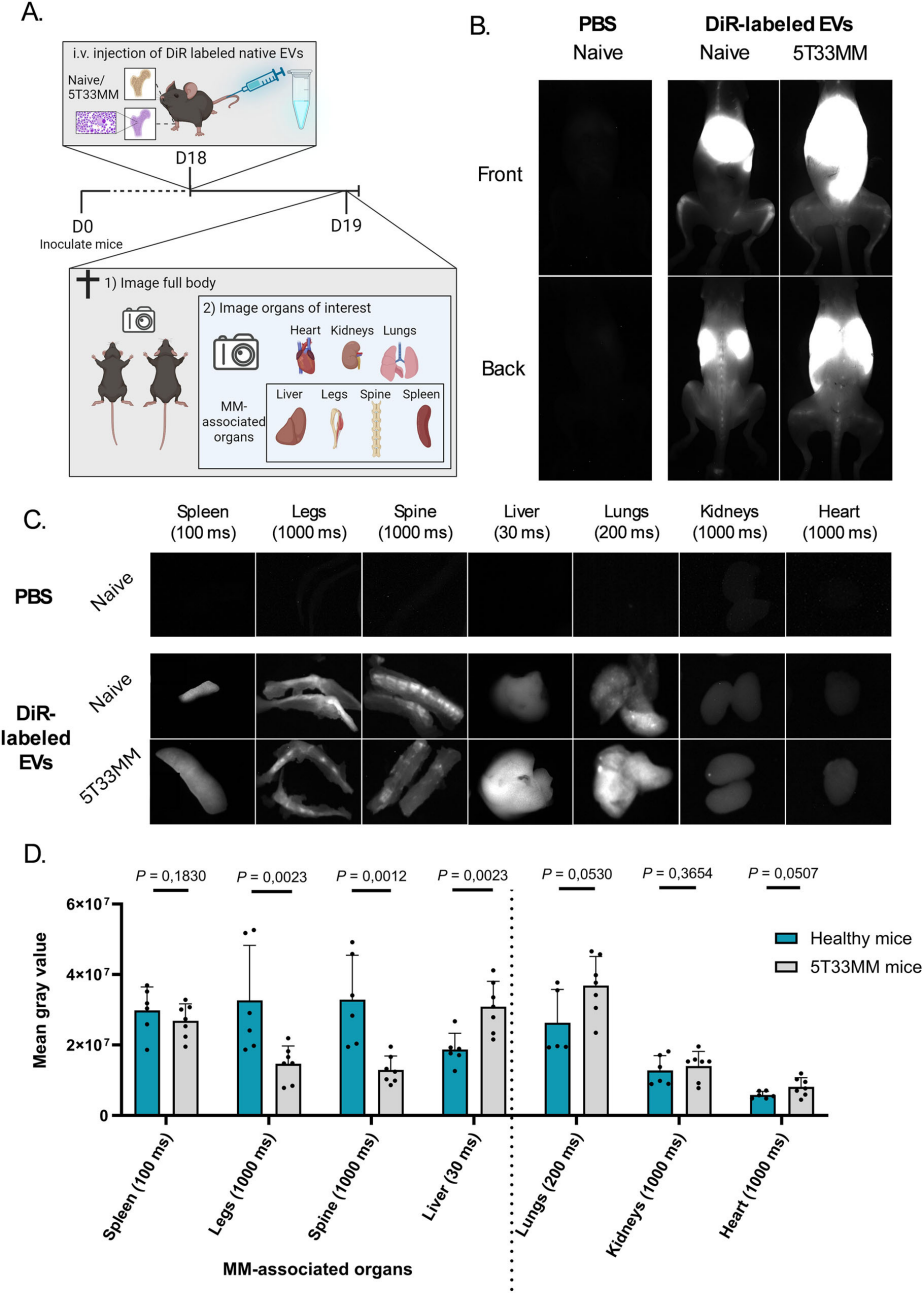
HEK293‐derived native EVs are retained more by the liver and distributed less to the legs and spine in myeloma‐bearing mice compared to healthy mice. (A) Experimental setup to compare the biodistribution of native EVs in naïve and MM‐bearing mice. Native EVs (9 × 10^10^, determined by Zetaview NTA) were labelled with DiR dye (5 µM), free dye was removed by ultracentrifugation and dyed EVs were injected in the tail vein of either healthy or 5T33MM mice 18 days post tumour inoculation. Twenty‐four hours post EV injection, the mice were sacrificed, skinned, imaged and dissected. (B) Full body images of naïve and 5T33MM mice 24 h after i.v. injection with the DiR labelled native EVs. Images were taken from the front and back of skinned mice using a Fluobeam 800 near‐infrared fluorescence camera. Exposure time for each image is 500 ms. Images from one mouse representative of seven 5T33MM and six healthy mice injected with DiR labelled native EVs and one naïve mouse injected with PBS to assess background fluorescence are shown. Note how the enlarged livers of 5T33MM mice are highly DiR^+^ compared to those of naïve mice. (C, D) Biodistribution of native EVs based on the DiR signal emitted from isolated organs. (C) Representative images from one mouse per group are shown. Exposure time is either maximum exposure (1000 ms) or the longest exposure with no saturation of the signal, as indicated. (D) MGVs quantified using ImageJ. Bars represent the mean ± SD of 7 5T33MM mice and 6 healthy mice. Statistical analyses were performed using a one‐tailed Mann–Whitney U‐test.

For therapeutic EVs to have an anti‐MM effect, they must reach the tumour sites, mainly being the liver, spleen and BM in the 5T33MM mouse model. Previously, we and others have shown that i.v. injected BM stromal cell (BMSC)‐derived EVs do reach the spleen, and to a lesser extent the BM in mice inoculated with 5T33MM tumour (Kang et al. 2021; Wang et al. 2014). However, no studies have evaluated the impact of MM disease on EV biodistribution. To evaluate this, non‐engineered HEK293‐derived EVs (native EVs) were isolated by ultracentrifugation and labelled with DiR. After removing the free dye by ultracentrifugation, native EVs were injected in naïve and end‐stage diseased 5T33MM mice (18 days post tumour inoculation). As negative controls, one naïve mouse injected with PBS and 3 naïve and 3 5T33MM mice injected with dye‐only preparations (DiR dye control) were also included. After 24 h, a time point at which we observed a clear signal in the hind legs for the EV conditions (Figure S1), the full body of the mice was imaged to visualize overall EV biodistribution (Figure 1(A–B)). Imaging revealed prominent signals in the upper body, particularly in MM‐bearing mice. In naïve mice, clear signals were also detected in the legs, whereas in 5T33MM mice, leg signals appeared weaker and displayed a more heterogeneous, patchy distribution, resembling the pattern of MM lesions (Vanderkerken et al. 1997). *Ex vivo* imaging of isolated organs provided objective measurements and largely confirmed these observations. EVs were significantly more distributed to the liver (3.1 ± 0.72 × 10^7^ mean grey value (MGV) vs. 1.9 ± 0.46 MGV, *p* = 0.0023) of 5T33MM mice compared to healthy mice (Figure 1(C–D)). Notably, EVs also tended to reach the lungs (3.7 ± 0.83 × 10^7^ MGV vs. 2.6 ± 0.94 × 10^7^ MGV, *p* = 0.0530) and heart (8.1 ± 2.6 × 10^6^ MGV vs. 5.8 ± 1.0 × 10^6^ MGV, *p* = 0.0507) more efficiently. Meanwhile, significantly less EVs were distributed to the legs (1.5 ± 0.51 × 10^7^ MGV vs. 3.3 ± 1.6 × 10^7^ MGV, *p* = 0.0023) and spine (1.3 ± 0.39 × 10^7^ MGV vs. 3.3 ± 1.3 × 10^7^ MGV, *p* = 0.0012), while EV distribution to the spleen (2.7 ± 0.48 × 10^7^ MGV vs. 3.0 ± 0.67 × 10^7^ MGV, *p* = 0.1830) and kidneys (1.4 ± 0.41 × 10^7^ MGV vs. 1.3 ± 0.42 × 10^7^ MGV, *p* = 0.3654) remained largely unchanged. These results indicate that in MM‐bearing mice, the distribution of EVs is significantly altered, providing the incentive to target the EVs towards the MM‐associated organs by, for example, displaying a Nb targeting the MM cell specific surface marker CS1.

### 3.2 Nanobodies Displayed on HEK293‐Derived EVs Using a Fusion Protein Based on Syndecan‐1

**FIGURE 2:**
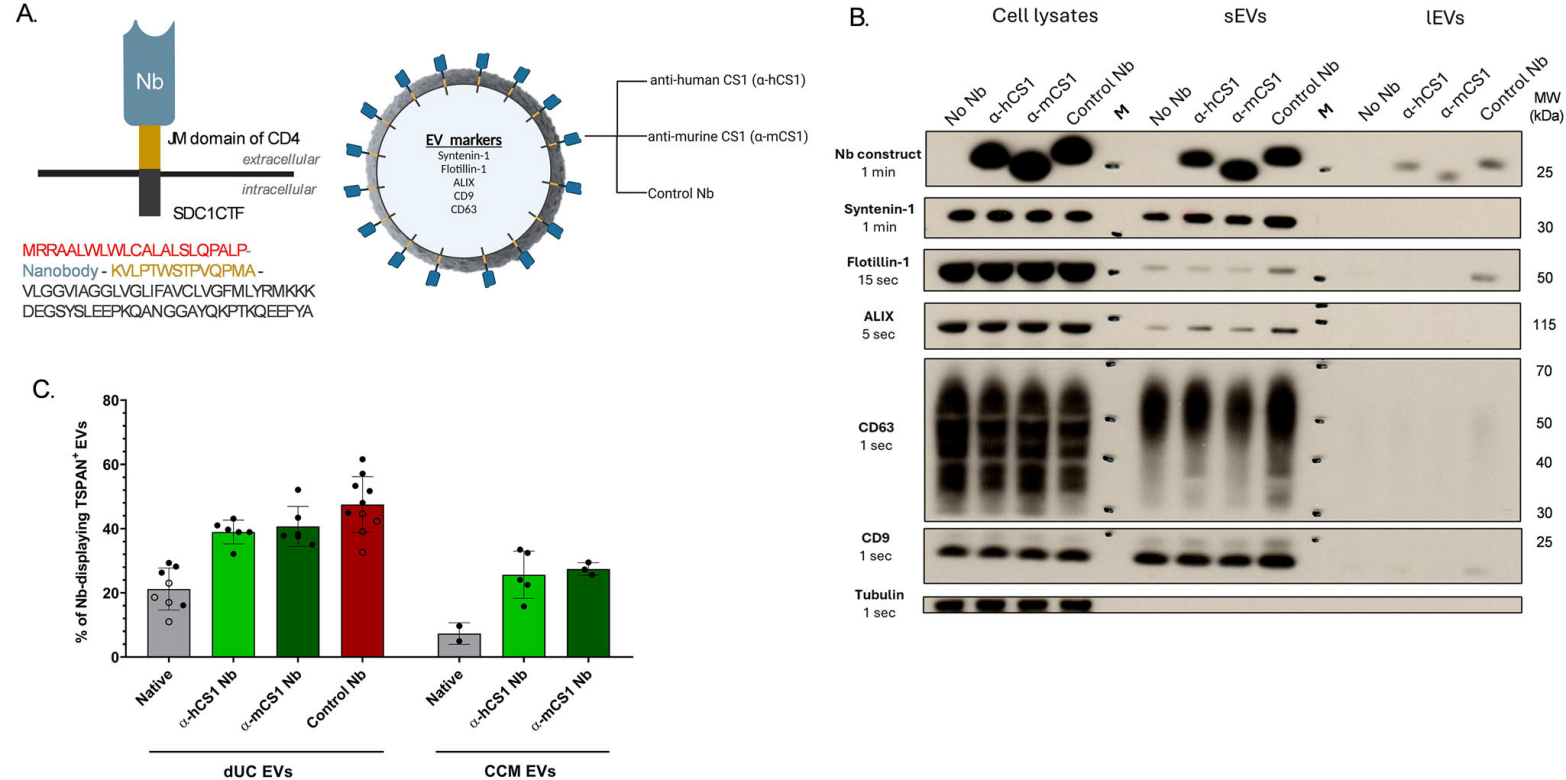
HEK293 cells stably transfected with Nb‐CD4JM‐SDC1CTF produce EVs displaying nanobodies. (A) Schematic representation of the Nb‐CD4JM‐SDC1CTF fusion proteins. (Left) Overview of the Nb‐CD4JM‐SDC1CTF fusion proteins designed for extracellular display of the Nb. The C‐terminal fragment of syndecan‐1 (SDC1CTF, black) is located intracellularly and integrates the fusion proteins into the syndecan‐syntenin EV biogenesis pathways. The N‐terminal Nb (blue) and C‐terminal SDC1CTF are linked by the juxtamembrane (JM) domain of CD4 (yellow) to prevent protease cleavage. (Right) HEK293 cells were stably transfected with Nb‐CD4JM‐SDC1CTF constructs, each with a different Nb: (1) anti‐human CS1 Nb (α‐hCS1 Nb), (2) anti‐murine CS1 Nb (α‐mCS1 Nb) and (3) control Nb R3B23 (control Nb). The latter has been extensively documented as an inert Nb in the 5T33MM mouse model used in this study (De Veirman et al. 2021; Hanssens et al. 2024; Lemaire et al. 2014). Native EVs are obtained from empty vector‐transfected cells. (B) Validation of Nb‐CD4JM‐SDC1CTF sorting into EVs. Stably transfected HEK293 cells and thereof derived isolated EVs were analysed for SDC1CTF (Nb construct) and the EV markers syntenin‐1, flotillin‐1, ALIX, CD63 and CD9 by western blot. Tubulin was used as a loading control (20 µg cell lysate (CL) protein). EVs from ∼1.5 × 10^6^ cells were loaded per well. Exposure times per target are constant between CLs, small EVs (sEVs) and large EVs (lEVs), as indicated. Indicated molecular weights correspond to the positions of the molecular weight markers on the blot. Data represent three independent experiments. Uncropped blots and quantifications of EV markers are provided in Figures S2 and S3, respectively. (C) Quantification of Nb‐displaying EVs. Graph showing the percentage of Nb‐displaying EVs of the total tetraspanin‐positive EV population. EVs were prepared by differential ultracentrifugation (dUC) or by concentrating conditioned medium (CCM). Overall EV population was determined by using pan‐tetraspanin antibodies (recognizing CD63, CD81 and CD9). Nb‐displaying EVs were determined using anti‐tetraspanin and anti‐Nb antibodies. EVs were immobilized on coverslips by phosphatidylserine binding and imaged using a super‐resolution Nanoimager (ONI). Images were analysed with CODI software (ONI). Data points represent technical repeats (from a single slide) from biological repeats (EVs produced independently, filled or empty circles). Bars show mean ± SD. Representative dSTORM images are shown in Figure S4. Note that although dUC and CCM native EVs show varying false positive signals due to prevalent non‐specific binding of anti‐V_HH_ antibodies (Muyldermans 2013), the relative Nb‐display remains consistent.

To display Nbs targeting CS1 on the surface of the HEK293‐derived EVs, we constructed fusion proteins consisting of an N‐terminal Nb fused to the C‐terminal fragment of syndecan‐1 that spans the transmembrane and cytosolic domain of the protein, via the cleavage‐resistant juxtamembrane domain of CD4 (Nb‐CD4JM‐SDC1CTF fusion proteins) (Fitzgerald et al. 2000). Overexpression of this fusion protein enables cleavage‐resistant extracellular display of a Nb (Figure 2(A), left) (Baietti et al. 2012; Hyka et al. 2026). We created three stably transfected HEK293 cell lines expressing a Nb‐CD4JM‐SDC1CTF fusion protein, each with a different Nb: (1) an anti‐human CS1 Nb (α‐hCS1 Nb), (2) an anti‐murine CS1 Nb (α‐mCS1 Nb) and (3) a control Nb, R3B23, binding an irrelevant epitope and serving as control for CS1‐independent effects of Nb display on EVs (Figure 2(A), right) (Puttemans et al. 2022). R3B23 has no binding reactivity in the 5T33MM model and is rapidly cleared by glomerular filtration in its soluble form (De Veirman et al. 2021; Hanssens et al. 2024; Lemaire et al. 2014). HEK293 cells stably transfected with the empty vector serve as control, producing native EVs.

To evaluate the overexpression of the fusion proteins and the sorting to EVs, cell pellets, sEV enriched (pelleted at 100,000 × g) and lEV enriched (pelleted at 10,000 × g) fractions derived from HEK293 cells stably transfected with the fusion proteins were isolated by differential ultracentrifugation and analysed for the presence of SDC1CTF fusion protein and several EV markers. As shown in Figure 2(B), the fusion proteins were mainly retrieved in sEVs fractions. Characterization of the EV markers ALIX, flotillin‐1, CD63, syntenin‐1 and CD9 revealed no major changes in both sEVs and lEVs upon overexpression of the fusion proteins (Figure 2(B); Figures S2–S3).

To estimate the fraction of secreted EVs that display a Nb at the surface, native, α‐hCS1 Nb, α‐mCS1 Nb and control Nb EVs were immobilized on a cover slip and analysed using an ONI super‐resolution Nanoimager. Clusters of at least 4 positive signals of the pan‐tetraspanin (recognizing CD63, CD81 and CD9) antibody in a 150 nm diameter were considered as EVs, while the co‐occurrence of at least 4 positive signals of the pan‐tetraspanin antibody and at least 2 positive signals of the anti‐V_HH_ antibody were considered as Nb‐displaying EVs (Figure S4). In the EV preparations isolated by differential ultracentrifugation (dUC EVs), 17.76% ± 7.510% of α‐hCS1 Nb EVs, 19.49% ± 9.025% of α‐mCS1 Nb EVs and 26.33% ± 10.86% of control Nb EVs were displaying a Nb (Figure 2(C), left), when taking the number of false‐positive native EVs (21.19% ± 6.53%) into account. For EVs prepared by concentrating conditioned medium (CCM EVs), 18.31% ± 8.096% and 20.12% ± 3.876% of the EVs were displaying α‐hCS1 Nbs and α‐mCS1 Nbs after subtracting the false‐positive fraction found in native EVs (7.32% ± 3.35%), respectively (Figure 2(C)). Overall, these results show that the overexpression of Nb‐CD4JM‐SDC1CTF fusion proteins in HEK293 cells results in the display of a Nb on roughly 20% of the EVs, regardless of the isolation method. In conclusion, overexpression of Nb‐CD4JM‐SDC1CTF fusion proteins in HEK293 cells has minimal impact on *bona fide* EV markers and is an efficient strategy to decorate the surface of their EVs with anti‐CS1 Nbs.

### 3.3 EVs Displaying αCS1 Nanobodies Bind Soluble CS1

**FIGURE 3:**
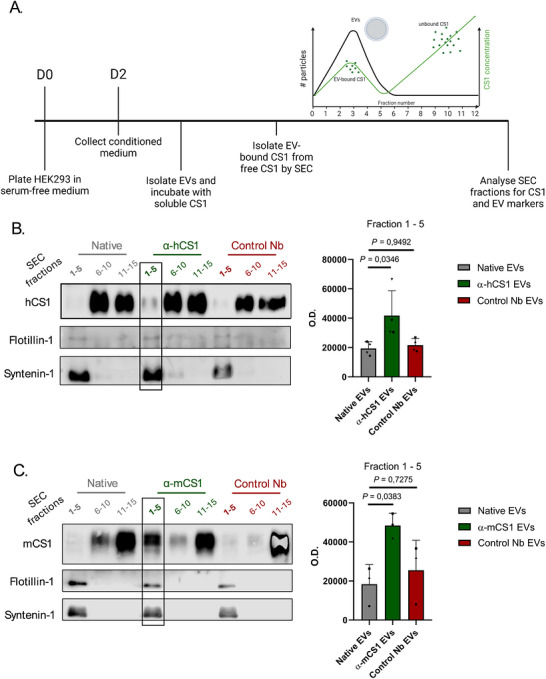
The α‐hCS1 and α‐mCS1 Nbs displayed on HEK293‐derived EVs bind to soluble recombinant CS1. (A) Experimental setup to determine the binding of the α‐CS1 Nb displaying EVs to soluble CS1. EVs (1 × 10^10^/mL, determined by Zetaview NTA) isolated by size‐exclusion chromatography (SEC) were incubated with 20.8 nM (resulting in a ratio of 1000 CS1 molecules per EV) soluble CS1 for 1 h. Next, free CS1 was separated from EVs by SEC and fractions 1–5, 6–10 and 11–15 were pooled, concentrated and analysed by western blot for the presence of CS1 and the EV markers flotillin‐1 and syntenin‐1. (B, C) Western blot analysis of the different SEC fractions of native, α‐CS1 Nb and control Nb EVs after incubation with soluble CS1. α‐hCS1 Nb (B) and α‐mCS1 Nb (C) displaying EVs co‐isolated with recombinant human (hCS1) and murine CS1 (mCS1), respectively. Left: one result representative of at least three independent experiments is shown. Note how fractions 1–5 of α‐CS1 EVs have a higher signal for CS1 compared to fractions 1–5 of native EVs and control Nb EVs. Right: Optical density (O.D.) of the CS1 bands for the pooled fractions 1–5. Bars represent mean ± SD of 4 (α‐hCS1 Nb) and 3 (α‐mCS1 Nb) independent experiments. Symbols indicate results obtained from separate experiments. Statistical analysis was performed using a one‐way ANOVA with Tukey's multiple comparisons test. Uncropped western blots are provided in Figure S6.

To validate the binding properties of the α‐CS1 Nbs displayed on the EV surface, engineered EVs were co‐incubated with soluble human recombinant CS1 in case of α‐hCS1 EVs, or murine recombinant CS1 for α‐mCS1 EVs, and size‐exclusion chromatography (SEC) was performed to separate the EV‐bound CS1 from the free, soluble CS1 (Figure 3(A)). Fractions 1–5, containing the majority of EVs (Figures S5–S6), and fractions 6–10 and 11–15, where we expect free soluble CS1 to elute, were pooled. As shown in Figure 3(B–C), we detected higher CS1 levels in the pooled fractions 1–5 of α‐hCS1 Nb EVs, with a 2.08‐ and 1.87‐fold average increase compared to native and control Nb EVs, respectively. Similarly, pooled fractions 1–5 of α‐mCS1 Nb EVs contained on average 2.64‐ and 1.90‐fold more CS1 than the native and control Nb counterparts. These findings provide evidence that the α‐CS1 Nbs displayed on the EV surface remain functional.

### 3.4 α‐mCS1 EVs Show Increased Retention in the Lungs of 5T33MM Mice

**FIGURE 4:**
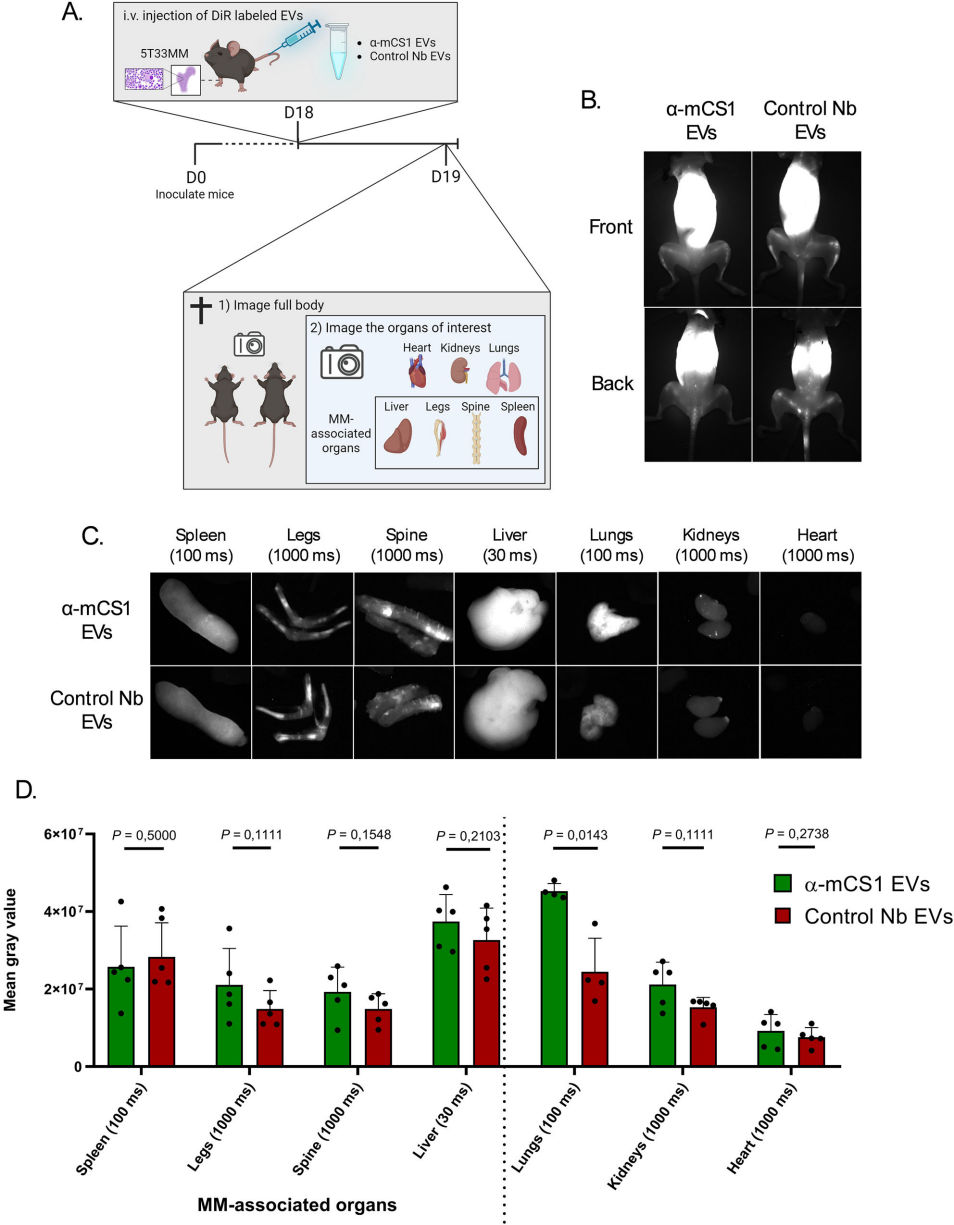
α‐mCS1 Nb EVs are retained more in the lungs compared to control Nb EVs in myeloma‐bearing mice. (A) Experimental setup to compare the biodistribution of α‐mCS1 Nb and control Nb EVs in MM‐bearing mice. α‐mCS1 Nb and control Nb EVs (9 × 10^10^, determined by Zetaview NTA) were labelled with DiR (5 µM). After removing free dye by ultracentrifugation, EVs were injected into the tail vein of 5T33MM mice 18 days post tumour inoculation. Twenty‐four hours after i.v. injection, the mice were sacrificed and skinned and the overall biodistribution of the EVs was determined using the Fluobeam 800 near‐infrared fluorescence camera. (B) Full body images of mice 24 h after i.v. injection with α‐mCS1 Nb and control Nb EVs. Images were taken from the front and back of skinned mice using a Fluobeam 800 near‐infrared fluorescence camera. Exposure times for images shown is 500 ms. Images from one mouse representative of 5 mice injected with α‐mCS1 Nb or control Nb EVs are shown (*n* = 5/group). Note that α‐mCS1 Nb EVs show no immediate differences in biodistribution compared to control Nb EVs. (C, D) *Ex vivo* determined biodistribution of α‐mCS1 Nb and control Nb EVs based on the DiR signal from isolated organs. (C) Images from one representative mouse are shown. Exposure time is either maximum exposure (1000 ms) or the longest exposure with no saturation of the signal, as indicated. (D) MGVs for all mice quantified using ImageJ. Bars represent the mean ± SD of *n* = 5, except for the lungs where *n* = 4, as the signal of the lungs of one mouse receiving anti‐CS1 Nb EVs was disturbed by clotted blood. Statistical analyses were performed using a one‐tailed Mann–Whitney U‐test.

To evaluate whether the display of an α‐CS1 Nb on the surface of the HEK293 EVs impacts their biodistribution, α‐mCS1 Nb or control Nb EVs were labelled with DiR and i.v. injected in end‐stage diseased 5T33MM mice. Mice were imaged after 24 h to determine overall EV biodistribution (Figure 4(A)). At 300 ms of exposure, a strong DiR signal was detectable in the liver and spleen regions in both conditions (Figure 4(B)). Moreover, in both groups, the front images revealed a clear signal emitted from the legs, while back images showed a clear signal in the legs and spine. Overall, on a whole‐body level, display of the α‐mCS1 Nb did not noticeably alter EV biodistribution compared to the control Nb.

To test for potentially more subtle differences in EV biodistribution, organs were dissected and imaged (Figure 4(C)), and the MGVs of the excised organs were quantified (Figure 4(D)). In line with the whole‐body imaging results, a DiR signal was predominantly seen in the liver, lungs and spleen. Interestingly, α‐mCS1 EVs were significantly more distributed to the lungs (4.5 ± 1.9 × 10^7^ MGV vs. 2.4 ± 0.87 × 10^7^ MGV, *p* = 0.0143) compared to control Nb EVs. Mice injected with α‐mCS1 EVs also showed a stronger average DiR signal in the liver (3.7 ± 0.70 × 10^7^ MGV vs. 3.2 ± 0.82 × 10^7^ MGV, *p* = 0.2103), spine (1.9 ± 0.64 × 10^7^ MGV vs. 1.5 ± 0.39 × 10^7^ MGV, *p =* 0.1548), legs (2.1 ± 0.94 × 10^7^ MGV vs. 1.5 ± 0.47 × 10^7^ MGV, *p* = 0.1111) and kidneys (2.1 ± 0.57 × 10^7^ vs. 1.5 ± 0.25 × 10^7^ MGV, *p* = 0.1111) compared to control Nb EVs, but these differences were not significant. Importantly, control Nb EVs had a comparable biodistribution profile compared to native EVs (Figure S8), confirming once again that R3B23 is an appropriate Nb control for *in vivo* studies in 5T33MM mice. To conclude, in 5T33MM mice, display of an α‐mCS1 Nb on HEK293‐derived EVs helps to modestly increase their retention by the MM‐associated organs (including legs, spine and liver), but is also accompanied by a significant increase in the lungs compared to control Nb EVs.

### 3.5 Α‐mCS1 Nb EVs Have No Improved *In Vivo* Specificity for MM Cells

**FIGURE 5:**
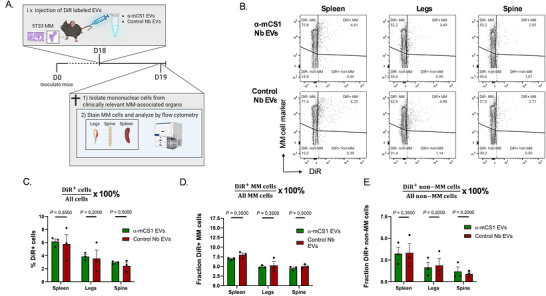
Display of the α‐mCS1 Nb on HEK293‐derived EVs does not improve specificity towards MM cells. (A) Experimental setup used to investigate cellular specificity of EV uptake in myeloma bearing mice. To investigate EV uptake by MM and non‐MM cells, native, α‐mCS1 Nb displaying and control Nb displaying EVs (9 × 10^10^, determined by Zetaview NTA) were labelled with DiR (5 µM). Free dye was removed by ultracentrifugation and EVs were i.v. injected in the 5T33MM mice 18 days post tumour inoculation. Twenty‐four hours later, cells were isolated from the spleen, legs and spine, and stained with the 3H2 antibody staining the 5T33MM idiotype antigen (MM cell marker). DiR fluorescence in both MM and non‐MM cells was assessed by flow cytometry. (B–E) EV uptake by the MM and non‐MM cells in the spleen, legs and spine 24 h after i.v. injection. (B) Flow cytometry data of one representative experiment, showing cells positive for the anti‐idiotype antibody on the *y*‐axis and cells positive for the DiR dye on the *x*‐axis. Gating depended on the FMOs is shown in Figure S9A. (C–E) The % DiR^+^ cells (C), the fraction of DiR^+^ MM cells (D) and the fraction of DiR^+^ non‐MM cells (E). Bars represent the mean ± SD of three independent experiments. Statistical analysis was performed using a one‐tailed Mann–Whitney U test. The tumour burden for each mouse is provided in Figure S9.

As EVs are clearly able to the reach the MM‐bearing organs, we next investigated whether display of an α‐mCS1 Nb confers increased specificity of the EVs towards MM cells in these organs. Cells from the spleen, legs and spine of the 5T33MM mice injected with DiR‐labelled α‐mCS1 Nb or control Nb EVs, were isolated and stained for a tumour‐specific marker expressed by 5T33MM cells (namely the 5T33 idiotype (= MM cell marker)). Next, EV delivery to MM and non‐MM cells was analysed by flow cytometry (Figure 5(A)). In general, more DiR^+^ cells (± 6%) were observed in the spleen of mice treated with either EV type compared to the legs (± 4%) and spine (± 3%) (Figure 5(B–C)). This was true for both MM (Figure 5(B–D)) and non‐MM cells (Figure 5(B), (E)). No notable differences were found between α‐mCS1 Nb and control Nb EVs in either MM (Figure 5(D)) or non‐MM cells (Figure 5(E)) across all investigated organs. Across all EV types, no differences in the percentage of DiR^+^ cells were observed in the spleen and spine (Figure S9). In the legs, both α‐mCS1 Nb and control Nb EVs showed a decrease in DiR^+^ cells compared to native EVs. This reduction was primarily driven by a lower percentage of DiR^+^ MM cells, while the percentage of DiR^+^ non‐MM cells remained comparable to native EVs. The tumour burden of the mice at the time of imaging is shown in Figure S9F. Overall, the display of an α‐mCS1 Nb on EVs does not improve the specificity of the HEK293 EVs towards MM cells in the 5T33MM model.

### 3.6 A Dose‐Escalation Study Reveals Saturation of Liver, Spleen and Lungs Predominantly at the Full Dose

To better understand dose‐dependent biodistribution and off‐target accumulation, we next performed a dose‐escalation experiment using DiR‐labelled α‐mCS1 Nb EVs in 5T33MM diseased mice, administrating 3 different doses (full, half and quarter dose). After 24 h, organs were dissected, imaged and the MGVs were again quantified. As expected, a clear dose‐dependent signal was observed in the liver, spleen and lungs (Figure S10). Notably, the DiR signal in the liver increased 26.85% when comparing quarter to half dose, while the signal only rose 4.95% when comparing half to full dose. Similar observations were made for the spleen (39.89% increase for quarter to half dose and 7.98% increase for half to full dose) and the lungs (26.10% increase for quarter to half dose and 16.17% increase for half to full dose), implying that some saturation of these organs occurs at higher EV doses. In contrast, no marked dose‐dependent differences were seen in the other organs, including legs and spine.

Nevertheless, at the cellular level, a higher (albeit not significant) percentage of DiR^+^ MM cells was detected in the BM of the legs of mice that received the full dose (Figure S10). However, this dose‐dependent uptake in MM cells was not observed in the spleen or spine. Additionally, no dose‐dependent effects were observed for the non‐MM cells. Comparable results were observed when considering DiR^+^ CS1^+/−^ cells, confirming the validity of our results. Of note, MM burden and CS1^+^ cell populations were consistent among groups. Altogether, these findings suggest that once uptake by the liver, spleen and, to a lesser extent, lungs becomes saturated, EVs remain longer in circulation and are more likely to reach the BM of the legs.

### 3.7 EVs Displaying Nanobodies Show Increased Uptake by MM and Non‐MM Cells in *Ex Vivo* BM Cultures

**FIGURE 6:**
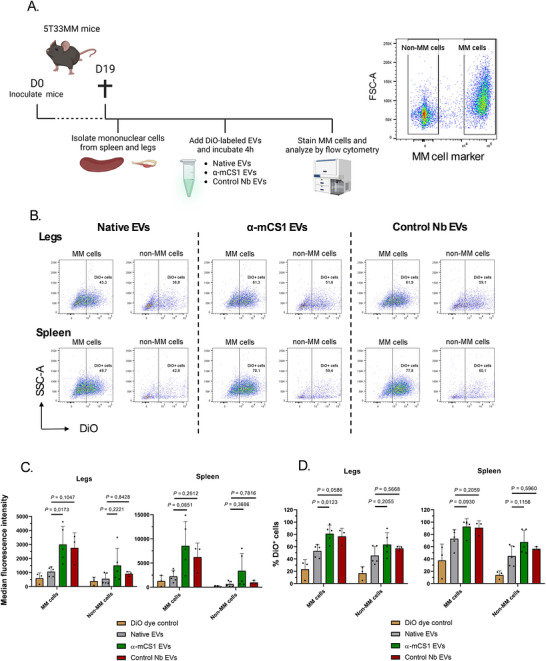
α‐mCS1 Nb EVs show increased EV uptake by leg‐derived MM cells compared to native EVs in *ex vivo* cultures. (A) Experimental setup and gating strategy to assess the specificity of α‐mCS1 displaying EVs towards MM cells. Nineteen days after tumour inoculation, 5T33MM mice were sacrificed and mononuclear cells were isolated from the spleen and BM of the legs. Native, α‐mCS1 Nb and control Nb EVs (3 × 10^10^, determined by Zetaview NTA) were labelled with DiO (5 µM), washed by ultracentrifugation and added to 1 × 10^5^ cells derived from the spleen and BM. Cells exposed to dye‐only preparations consisting of DiO‐labelled 5% EDS medium (DiO dye control) were also included. Four hours later, cells were stained for the MM idiotype, fixed and analysed by flow cytometry. (B–D) *Ex vivo* uptake of native, α‐mCS1 Nb and control Nb EVs by MM and non‐MM cells in cell cultures isolated from the BM and spleen. (B) Uptake of native, α‐mCS1 Nb and control Nb EVs by MM and non‐MM subpopulations isolated from the legs (up) and spleen (down) was measured by determining the % DiO^+^ cells. Results from one representative experiment are shown. (C) The median fluorescence intensity (MFI) for DiO and (D) the % DiO^+^ cells as measure for EV uptake by MM and non‐MM cells for multiple experiments. Mean ± SD is shown (DiO dye control: *n* = 3, native EVs: *n* = 5, α‐mCS1 Nb EVs: *n* = 5, control Nb EVs: *n* = 3). Statistical analyses were performed with a one‐way ANOVA with Tukey's multiple comparisons test.

Since we observed that the display of an α‐mCS1 Nb on EVs increases their retention by the liver, lungs and kidneys of 5T33MM mice, but fails to specifically target MM cells in the spleen, legs and spine, we next examined if display of an α‐mCS1 Nb can improve EV specificity to MM cells in *ex vivo* spleen and BM cultures. For this, mononuclear cells isolated from the legs and spleen of end‐stage diseased 5T33MM mice were isolated and incubated with DiO‐labelled native, α‐mCS1 Nb or control Nb EVs for 4 h (Figure 6(A). Next, the cells were stained for the 5T33 idiotype and the MFIs for DiO (Figure 6(C)) and the % DiO^+^ cells (Figure 6(B), (D)) were determined in the MM and non‐MM subpopulations. Both in the BM (Figure 6(C–D), left) and spleen (Figure 6(C–D), right) cultures, α‐mCS1 Nb and control Nb EVs tend to be better taken up by both the MM and non‐MM cells compared to native EVs. This increased uptake was only significant for the α‐mCS1 Nb EVs in the MM cells of the BM cultures, with a 1.53‐fold higher uptake compared to native EVs (*p* = 0.0123). Interestingly, all cells derived from the spleen, particularly the MM cells, exhibited a stronger DiO signal than the BM‐derived cells when treated with any DiO‐labelled EV type (Figure 6(C)), suggesting higher basal EV uptake in the spleen‐derived populations. Taken together, although the display of either an α‐mCS1 Nb or a control Nb on EVs enhanced EV uptake in both cell subpopulations, display of the α‐mCS1 Nb resulted in slightly more DiO^+^ MM cells compared to native and control Nb EVs, especially in leg BM cultures.

### 3.8 α‐hCS1 Nb EVs Show Enhanced Uptake by CS1+ Human Myeloma Cell Monocultures

**FIGURE 7:**
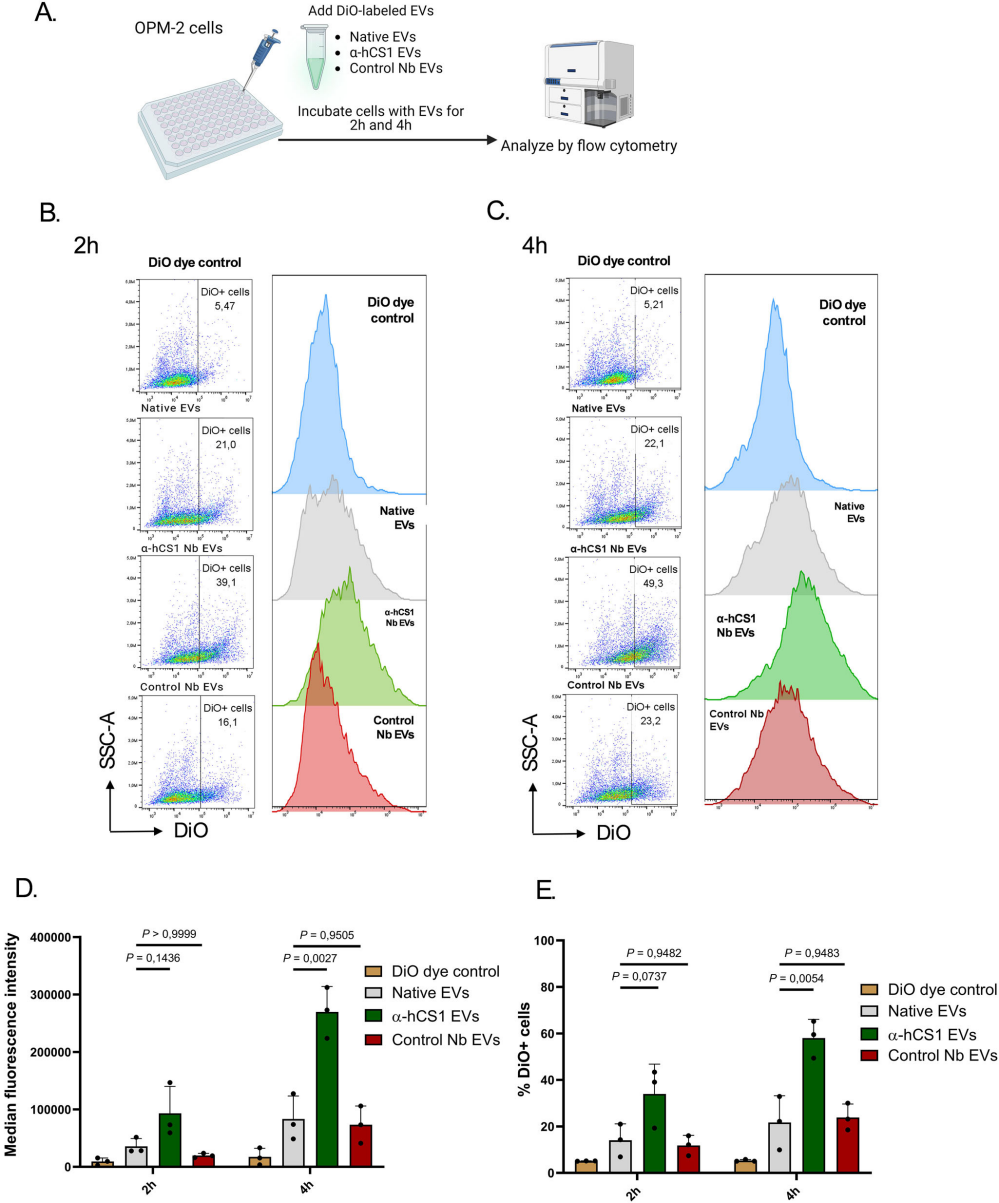
The uptake of α‐hCS1 Nb‐displaying EVs by human myeloma cells is enhanced compared to native and control Nb EVs. (A) Experimental setup to determine EV uptake of DiO‐labelled native, α‐mCS1 Nb and control Nb EVs by CS1^+^ human OPM‐2 MM cells. Native, α‐hCS1 Nb or control Nb displaying EVs (9 × 10^9^, determined by Zetaview NTA) were dyed with DiO (5 µM). Free dye was removed by ultracentrifugation and EVs were incubated with 50,000 CS1^+^ OPM‐2 cells for 2 h and 4 h. Cells exposed to dye‐only preparations consisting of DiO‐labelled 5% EDS medium (DiO dye control) were also included. Next, DiO fluorescence was measured by flow cytometry. (B, C) Dotplots and histograms of one representative experiment. Dotplots (left) and overlays of the histograms (right) of cells treated with DiO dye control (blue), native (grey), α‐hCS1 Nb‐displaying (green) and control Nb EVs (red) for 2 h (B) and 4 h (C). (D, E) Graphs showing the uptake of native, α‐hCS1 Nb and control Nb EVs by CS1^+^ human myeloma cells over multiple experiments. The median fluorescence intensity (MFI) for DiO (D) and the % DiO^+^ cells (E) are shown. Data are the mean ± SD of three independent experiments. Statistical analysis was performed with a one‐way ANOVA with Tukey's multiple comparisons test.

The results described above show that although the display of an α‐mCS1 Nb on EVs slightly improves their uptake by MM cells *in vitro*, improved MM specificity could not be shown *in vivo*, at least not in the present experimental setting. These findings advocate for an intratumoral injection approach, where EVs are directly injected in a palpable tumour of CS1^+^ human myeloma cell line that is strongly positive for CS1 (Figure S11) and eligible for xenografting (Vlummens et al. 2019), such as OPM‐2. To test if this approach could be viable for future testing, we investigated if display of an α‐hCS1 Nb on HEK293‐derived EVs influences their uptake by an *in vitro* monoculture of OPM‐2 cells. DiO‐labelled native, α‐hCS1 Nb and control Nb EVs were incubated with OPM‐2 cells for 2 h and 4 h after which the MFI and % DiO positive cells were determined using flow cytometry (Figure 7(A)). The MFI for α‐hCS1 EVs was on average 2.60 times higher after two hours and 3.24 times higher after four hours compared to the native EVs and 4.71 times and 3.67 times compared to the control Nb EVs respectively (Figure 7(B)–(D)). In terms of DiO^+^ cells, 33.93% ± 12.85% of the OPM‐2 cells treated with α‐hCS1 EVs were found DiO^+^ after 2 h of incubation, while cells treated with native and control Nb EVs were 14.07% ± 7.043% and 11.83% ± 4.31% DiO^+^, respectively (Figure 7(B), (E)). EV uptake was further increased after 4 h (Figure 7(C), (E)), with 58.03% ± 8.00% of the cells being DiO^+^ when treated with α‐hCS1 EVs compared to 21.67% ± 11.50% and 23.90% ± 5.78% for the native and control Nb EVs, respectively. These results indicate that display of α‐hCS1 Nb does facilitate EV uptake by a monoculture of human CS1^+^ MM cells compared to native EVs and EVs displaying a control Nb.

## 4 Discussion

EV therapeutics are under thorough preclinical and early clinical investigation, particularly for cancer treatment (Zhang et al. 2021). EVs derived from a variety of cell types, including MSCs, fibroblasts, various immune cell types, tumour cells and HEK293 cells, are loaded with chemotherapeutic drugs, gene editing components or immune modulators, aiming for delivery to the tumour (Qian et al. 2022). Decorating these EVs with targeting moieties is an approach to increase tumour targeting and sparing healthy tissue (Gupta et al. 2023), which was already explored in the context of MM (Yuan et al. 2024). Yuan et al. reported enhanced myeloma cell killing, anti‐myeloma immunomodulation and increased inhibition of MM‐induced osteolysis after treatment of myeloma‐bearing mice with bortezomib‐loaded BCMA‐targeting monocyte‐derived EVs compared to bortezomib treatment alone. However, despite these advancements, significant gaps remain in understanding how disease‐specific contexts, such as MM and cancer in general, influence EV biodistribution. Furthermore, previous research mainly focused on enhancing tumour‐specific targeting, while less attention has been paid to the broader implications of targeting moieties on overall EV biodistribution.

To address the underexplored aspect of possible MM‐related effects on EV biodistribution, this study first compared the biodistribution of native HEK293‐derived EVs in healthy and MM‐bearing mice. Additionally, we assessed the ability of HEK293‐derived EVs to reach the tumour sites, a key aspect in unlocking the therapeutic potential (Choi et al. 2024). We focused on comparing the distribution of HEK293‐derived EVs to the organs affected by the disease in the 5T33MM model, with particular attention to the clinically relevant sites of disease involvement in humans, being the legs and spine. In contrast, the liver and spleen of 5T33MM mice are also involved in the MM disease, but rarely harbour MM lesions in human patients (Bladé et al. 2022; Seo et al. 2011; Vanderkerken et al. 1997). HEK293 cells were selected as EV producers because of their ease and speed of culture, high transfection efficiency and clinical track record of producing biological therapeutics (Tan et al. 2021).

In line with previous findings, we found that all the organs that are affected by the MM disease in the 5T33MM model (the bone, liver and spleen) are reached by native HEK293‐derived EVs, both in naïve mice and 5T33MM mice (Kang et al. 2021; Wiklander et al. 2015), making them potentially interesting therapeutic vesicles for MM. However, we here show for the first time that the distribution of HEK293‐derived EVs to the primary human myeloma‐associated organ, namely the bone, is strongly impaired in 5T33MM diseased mice. A possible explanation for this observation could be the well‐documented presence of abnormal, distorted microvessels associated with MM (Hu et al. 2012; Van Valckenborgh et al. 2002), which can impair local blood circulation, a common phenomenon in cancer (Carmeliet and Jain 2011). This dysfunctional vasculature may hamper efficient EV distribution to the bone. A second explanation for the reduced delivery to the legs and spine may be that the lungs and liver act as sinks that capture EVs, especially since these organs are the first major filters that compounds encounter upon i.v. injection (Xie et al. 2010; Yoo et al. 2010). Indeed, we observed increased EV retention in the liver and lungs of 5T33MM mice compared to naïve mice. While the liver is naturally a primary site of EV uptake due to its mononuclear phagocyte system (MPS) (Cieślik et al. 2023), this retention seems amplified in the MM context, potentially due to organ enlargement because of the massive myeloma cell infiltration. Indeed, previous literature shows how cancerous B cells (which are the same lineage as myeloma cells) promptly take up more EVs than non‐cancerous B cells (Hazan‐Halevy et al. 2015). Another possible explanation is the changes in macrophage activity induced by the disease, as our group previously showed an increase in M2‐polarized macrophages in 5T33MM mice during MM progression (De Beule et al. 2017), which are associated with increased phagocytosis (Jaggi et al. 2020). Similarly, the lungs show heightened EV accumulation, which may be the result of disease‐induced inflammation or lung plasmacytosis, as both have been documented in human MM patients (Kintzer 1978). Altogether, these findings demonstrate that the distribution of i.v. injected HEK293‐derived EVs is altered in MM‐bearing mice, underscoring the challenge of ensuring EVs reach the MM‐affected sites like the BM.

This prompted us to explore strategies to enhance the tumour specificity of the HEK293‐derived EVs. By overexpressing Nb‐CD4JM‐SDC1CTF fusion proteins in HEK293 cells, we engineered EVs that display an α‐CS1 Nb on their surface. We showed that a substantial fraction (±20%) of the EVs secreted by cells stably expressing Nb‐CD4JM‐SDC1CTF successfully display the Nb on their surface, ensuring the presence of a clear Nb‐displaying EV population. This fraction of 20% is comparable to a GPI‐anchor‐mediated approach for Nb display (Kooijmans et al. 2016), but seems to underperform when compared to a sorting domain based on the N‐terminus of syntenin, which reached up to 40% of display efficiency (Gupta et al. 2021). Nevertheless, we confirmed that EVs produced by the anti‐CS1 Nb‐expressing cells effectively bind soluble CS1.

Next, we investigated the biodistribution and cellular uptake of the HEK293‐derived EVs engineered in relevant *in vitro* and *in vivo* MM models. When comparing the biodistribution of α‐mCS1 Nb EVs to control Nb EVs in 5T33MM mice, they slightly outperformed in reaching the clinically relevant tumour sites. Importantly, native and control Nb EVs exhibited similar biodistribution profiles, reinforcing the validity of R3B23 as control Nb. Additionally, greater retention of the α‐mCS1 Nb EVs was also observed in the liver, kidneys and especially the lungs. This enhanced retention in the liver was not surprising, as the liver is, as mentioned above, a MM infiltrated organ in the 5T33MM model. Moreover, our group previously demonstrated how free α‐mCS1 Nb (also referred to as sdAb1) not only accumulates more in the legs and spine of MM diseased mice compared to the control Nb, but also in the spleen, liver and lungs (De Veirman et al. 2021). Hence, displaying an α‐mCS1 Nb on the EV surface may amplify their (off‐tumour) retention, with CS1^+^ cells in the liver and lungs potentially sequestering EVs and creating a sink effect that restricts distribution to the legs and spine. This notion is supported by the dose‐escalation data indicating partial saturation of liver, spleen and lungs uptake at higher EV doses, while against expectations, no increased retention of α‐mCS1 EVs was observed in the legs and spine.

However, although injected α‐mCS1 EVs did reach the bones and liver slightly better than the control Nb EVs, display of the α‐mCS1 Nb conferred no improvement in specificity toward MM cells over non‐MM cells *in vivo* compared to native or control Nb EVs. Flow cytometry analysis of cells from the spleen, legs and spine of 5T33MM mice injected with DiR‐labelled EVs revealed that a larger proportion of spleen cells exhibited DiR^+^ signals (±6%) than those from the legs (±4%) or spine (±3%), regardless of EV type. However, no notable differences in % DiR^+^ cells emerged between α‐mCS1 Nb and control Nb EVs for either MM or non‐MM cells across these organs. Moreover, in the legs, both α‐mCS1 Nb and control Nb EVs showed fewer DiR^+^ MM cells compared to native EVs. These findings indicate that while the α‐mCS1 Nb boosts organ‐level retention, it does not translate to improved cellular specificity *in vivo*, underscoring the difficulty of achieving precise MM cell targeting amidst systemic EV sequestration.

To further assess the cellular specificity of α‐mCS1 Nb EVs, we decided to circumvent the systemic EV sequestration by isolating BM and spleen cells and adding the EVs *ex vivo*. These *ex vivo* cultures of BM and spleen cells from 5T33MM mice revealed that both the α‐mCS1 Nb and control Nb enhanced EV uptake by MM and non‐MM cells compared to native EVs, with spleen‐derived cells, especially MM cells, showing stronger DiO signals than BM‐derived cells. These results suggest enhanced uptake of Nb‐CD4JM‐SDC1CTF engineered EVs, independent of the specificity of the Nb. Since SDC1CTF is the common denominator of both, it could provide a mechanistic explanation. Possibly, overexpression of SDC1CTF may influence the PDZ (post synaptic density protein, *Drosophila* disc large tumour suppressor, zonula occludens‐1 protein) network, as SDC1CTF carries a PDZ‐binding motif at its C‐terminus (Castro‐Cruz et al. 2023). SDC1CTF could possibly facilitate uptake of the EVs themselves or enhance endocytosis by the recipient cells. Importantly, in the human OPM‐2 MM cells, we did see an improved uptake of the α‐CS1 Nb EVs compared to the control Nb EVs, implying that this phenomenon is cell‐context dependent. This has to be studied more in depth but is beyond the scope of this study. Overall, our results suggest that CS1‐independent uptake mechanisms are prominent for our Nb engineered EVs in the current murine *ex vivo* coculture setting (Cerezo‐Magaña et al. 2020), while systemic factors dictate CS1‐dependent off‐target retention *in vivo* in the 5T33MM model.

While our study provides novel insights into EV targeting in MM and confirms the feasibility of our platform *in vitro*, inherent limitations of the current study restrict us to fully assess its *in vivo* applicability and ultimately its therapeutic potential. Firstly, while a substantial fraction (±20%) of the EVs display the Nb, the predominance of non‐targeted native EVs in our preparations, together with the use of generic lipophilic dyes for EV tracking, represents a key limitation of the current study. Lipid dyes, while commonly used to track EVs, are associated with dye aggregation, nonspecific membrane transfer and background signal (Gupta et al. 2023; Ripoll et al. 2026; Simonsen 2019). Hence, our targeted EVs may be overshadowed by a large population of DiO/DiR‐labelled αCS1 Nb‐negative EVs, reducing signal‐to‐noise ratios. To address this, future studies should focus on improving EV purity through affinity‐based enrichment strategies and incorporating a reporter, such as nanoluciferase or a fluorescent protein, in the Nb constructs, to enable direct and selective tracking of our engineered EVs. Secondly, given the substantial retention of α‐CS1 Nb EVs in the liver and lungs, further investigation is needed to determine where the EVs are located in these organs and whether they co‐localize with CS1 epitopes, which could clarify the mechanisms of off‐target or on‐target sequestration. In this regard, our *in vitro* findings with anti‐human CS1 Nb EVs, which demonstrated enhanced uptake by OPM‐2 human myeloma cells compared to native and control Nb EVs, provide a strong rationale for *in vivo* studies using an OPM‐2 xenograft model. Intratumoral injection of the EVs in this model could validate whether increased retention at the tumour site is achievable, circumventing systemic barriers and building on the promising uptake observed *in vitro*. Additionally, co‐display of two Nbs targeting distinct tumour antigens, such as BCMA and CS1, could be considered as a strategy to enhance targeting specificity. Thirdly, this study did not evaluate functional cargo delivery and/or therapeutic efficacy, which represent important yet challenging next steps. Increasing studies indicate that successful EV targeting and uptake do not necessarily translate into efficient functional cargo delivery, due to limited endosomal escape (Bonsergent et al. 2021; O'Brien et al. 2022). Accordingly, future studies should focus on elucidating the intracellular fate of our engineered EVs, focusing on internalization and endosomal escape, as well as on evaluating the functional activity of a therapeutic cargo. These future directions, alongside exploring alternative targeting moieties or delivery routes, will be crucial to overcoming the cellular and systemic sequestration challenges and advancing EV‐based therapies for MM.

Finally, although bioengineered EVs hold great therapeutic promise, their clinical translation remains hampered by both biological and technical limitations. From a biological perspective, our understanding of the molecular mechanisms supporting EV interactions with recipient cells and functional cargo delivery is still incomplete. Yet, in recent years, it has become increasingly clear that donor–recipient cell pairing plays a critical role in effective intercellular communication and should therefore be carefully considered when selecting cell sources for EV‐based therapeutics (Gupta et al. 2023; Ripoll et al. 2026). Additionally, although EVs are often considered less immunogenic than synthetic carriers, their immunogenicity can vary substantially depending on the cell source, isolation method and surface modifications introduced during engineering processes. Functionalized EVs, such as those equipped with targeting moieties or fusogenic proteins that enhance cargo delivery by promoting endosomal escape, may acquire altered biological properties that could elicit unintended immune responses in repeated dose‐settings (Ripoll et al. 2026; Xu et al. 2026). From a technical perspective, clinical translation is further constrained by significant scalability and manufacturing challenges. Large‐scale EV production required for clinical application typically relies on bioreactor‐based donor cell systems, whose phenotype and EV output can shift under long‐term and dynamic culture conditions, leading to variable yield and functionality. Moreover, commonly used isolation procedures and storage conditions may also compromise EV membrane integrity, leading to reduced functionality and altered biodistribution. Additionally, many EV engineering, isolation and purification strategies lack GMP‐compatible scalability, while intrinsic product heterogeneity complicates quality control, potency assessment and batch‐to‐batch consistency. However, recent advances in standardized, GMP‐compliant manufacturing show that these challenges are increasingly being addressed. For example, the integration of large‐scale bioreactors with automated ultrafiltration/diafiltration (UF/DF) processes and in‐process monitoring of donor cell phenotype now enables high‐yield, reproducible EV production (Humbert et al. 2025; Li et al. 2025). While practical and regulatory frameworks continue to evolve, ongoing efforts to standardize scalable, GMP‐compliant workflows demonstrate that the field is actively moving toward the practical solutions needed to bring EV‐based therapies to the clinic.

In conclusion, this study provides the first evidence that delivery of HEK293‐derived EVs to the legs and spine, key myeloma‐associated organs, is attenuated in the 5T33MM murine model. Engineering these EVs with α‐CS1 Nb‐CD4JM‐SDC1CTF fusion proteins enabled effective display of an α‐CS1 Nb on their surface, modestly enhancing retention in the legs and spine and substantially increasing uptake in the liver, lungs and kidneys. However, this modification failed to improve specificity toward MM cells *in vivo*, as systemic sequestration by CS1^+^ cells in off‐target organs likely restricted EV availability at tumour sites. Despite this limitation, our findings offer critical insights into how MM alters EV biodistribution and how targeting moieties influence their systemic fate in diseased states. Moreover, our observations underscore the complexity of achieving precise and effective EV delivery in cancer therapy, emphasizing the need for comprehensive strategies to positively influence biodistribution dynamics for optimal therapeutic outcomes.

## Author Contributions

**Michiel De Coster**: conceptualization, investigation, writing – original draft, formal analysis, methodology. **Lukas Hyka**: investigation, methodology, formal analysis. **Sophie De Cock**: investigation, methodology, formal analysis. **Sofie Meeussen**: investigation, methodology, formal analysis. **Zuowei Wang**: investigation, methodology, formal analysis. **Chenggong Tu**: writing – review and editing. **Sophie Hernot**: methodology, resources. **Nick Devoogdt**: writing – review and editing, resources. **Guido David**: writing – review and editing. **Kim De Veirman**: writing – review and editing. **Karin Vanderkerken**: writing – review and editing. **Eline Menu**: writing – review and editing. **Pascale Zimmermann**: conceptualization, funding acquisition, writing – review and editing, supervision, resources. **Elke De Bruyne**: conceptualization, investigation, funding acquisition, writing – original draft, writing – review and editing, methodology, supervision, resources, formal analysis.

## Funding

This work was supported by Kom Op Tegen Kanker under Exo‐RAS, KotK_VUB/2021/12786/1 and the VUB strategic Research Programme under SRP84. Michiel De Coster and Lukas Hyka were both supported by a predoctoral fellowship of FWO Vlaanderen under 1S94423N and 1SE2422N respectively.

## Ethics Statement

Animal housing, treatment and experimental designs were approved by the Ethical Committee for Animal Experiments of the Vrije Universiteit Brussel (CEP 23‐281‐8 and CEP 25‐281‐8).

## Conflicts of Interest

The authors report no conflicts of interest.

## Supporting information




**Supporting Information**: jev270325‐sup‐0001‐SuppMat.docx

## Data Availability

The data generated in this study are available upon reasonable request from the corresponding authors.
