# Supplementary material for: Characterizing the Fate of Anti‐CS1 Nanobody Displaying Extracellular Vesicles in Multiple Myeloma
Source: J Extracell Vesicles. 2026 Jun 17;15(6):e70325. doi: 10.1002/jev2.70325 (PMC13275992; doi:10.1002/jev2.70325)
Supplement: Supplementary file 1 — Supporting Information: jev270325‐sup‐0001‐SuppMat.docx [file JEV2-15-e70325-s001.docx]

**
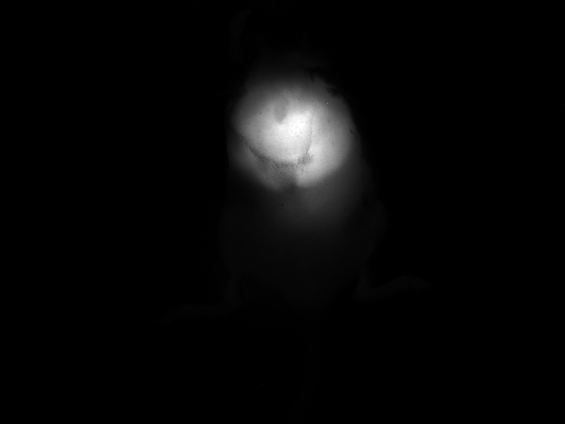

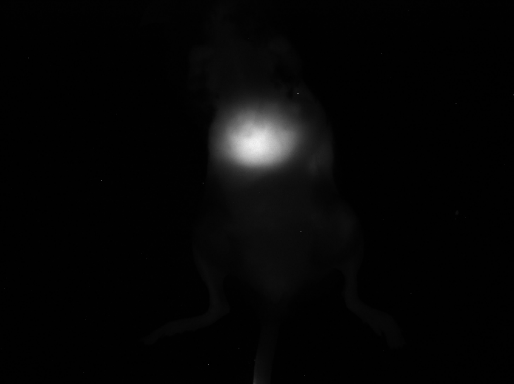

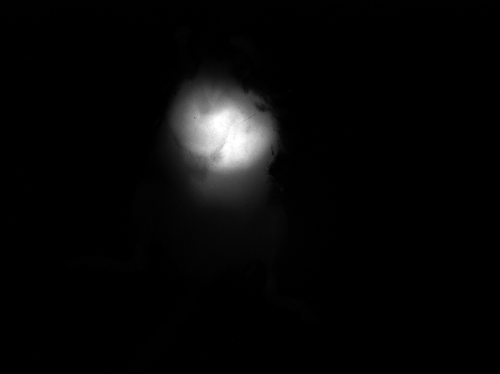

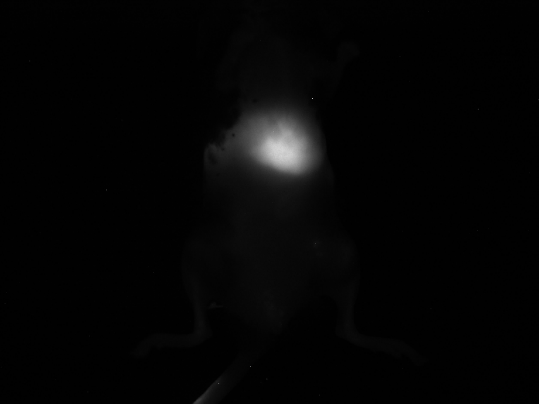

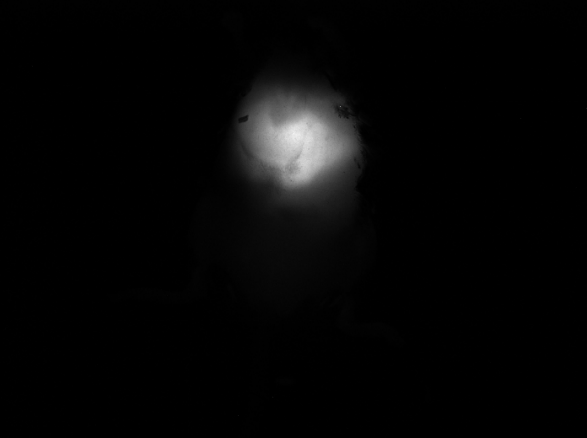

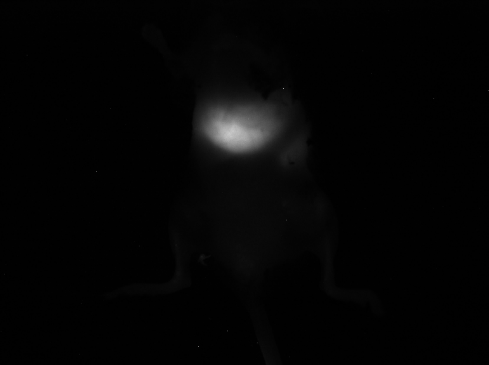

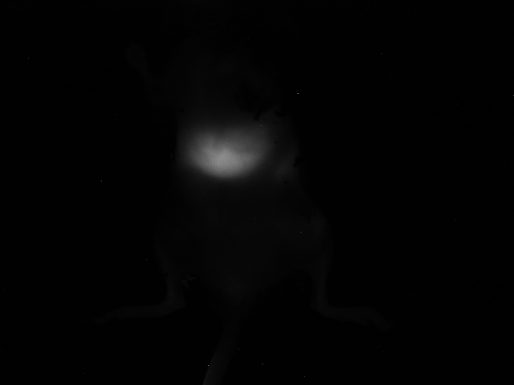

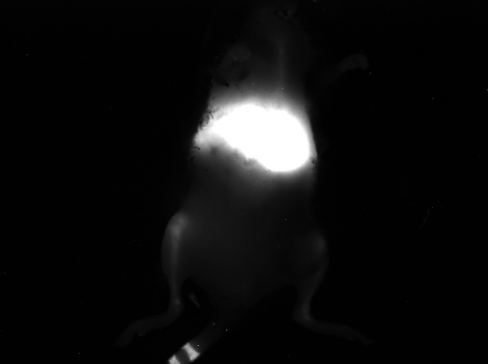

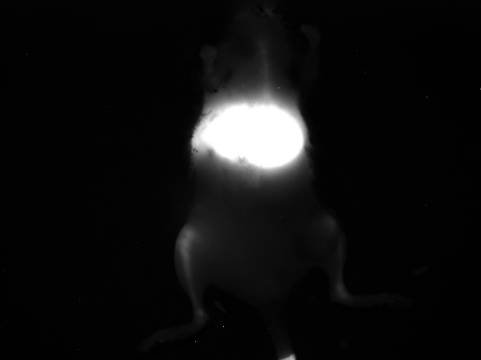

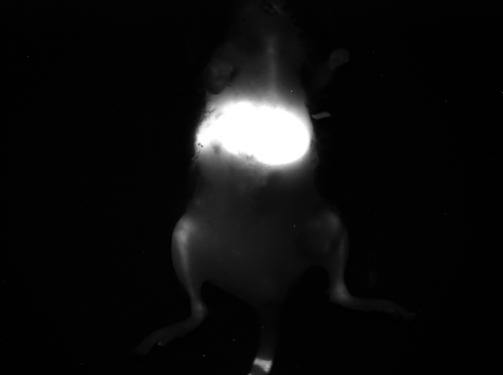

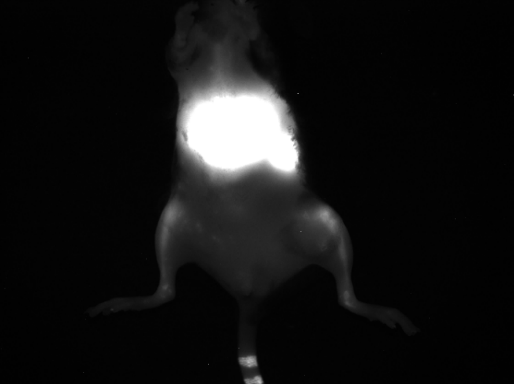

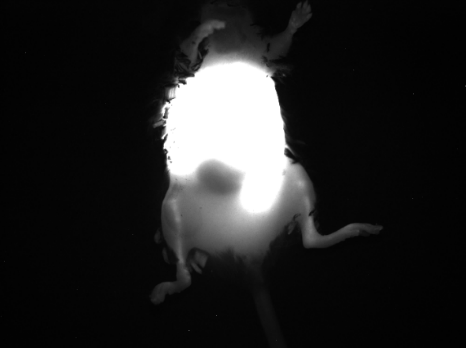

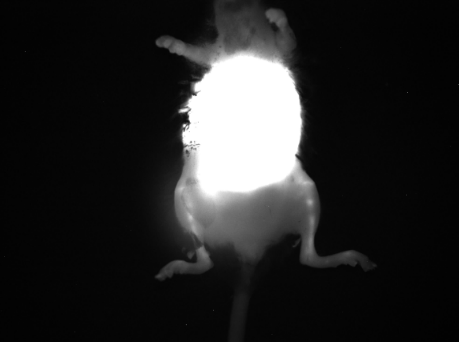

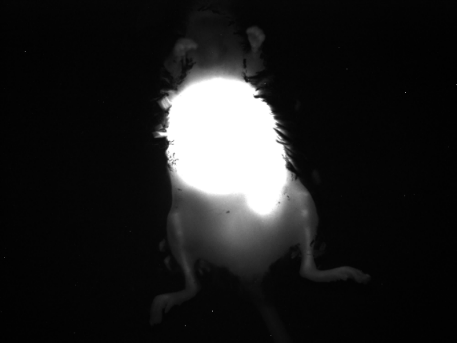

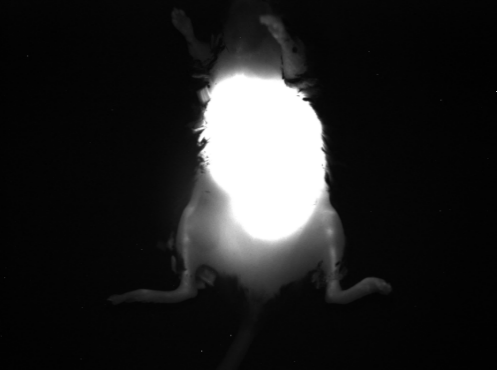
**

A.

Naive

**DiR dye control**

**DiR-labeled EVs**

t = 1h

t = 3h

t = 6h

t = 24h

5T33MM

5T33MM

Naive

**
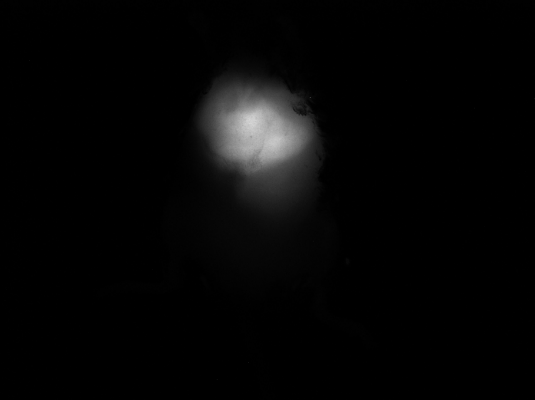
**


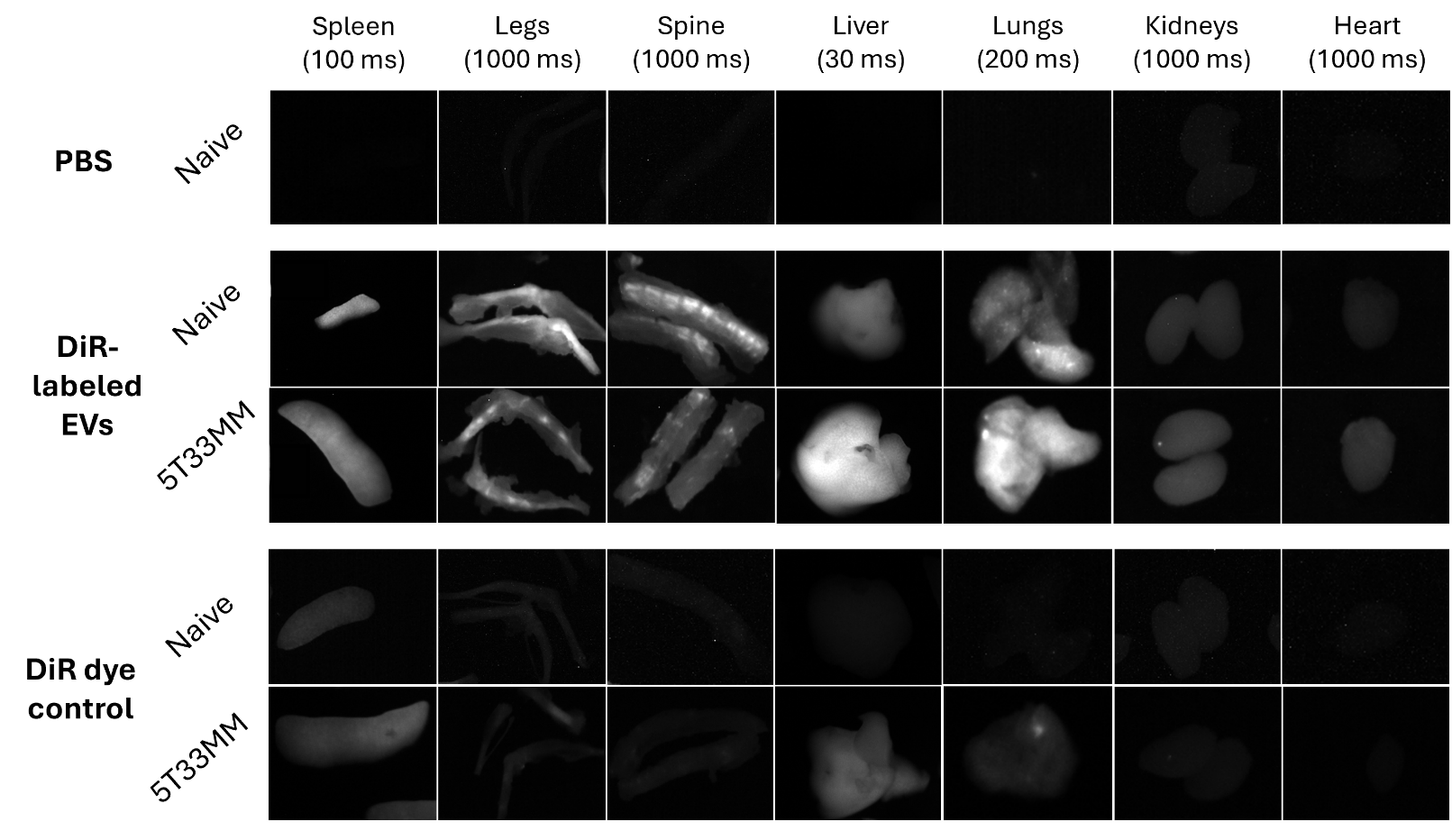


B.

C.

**Supplemental Figure S1: Comparing *in vivo* biodistribution of DiR-labelled native EVs and DiR dye control in naïve and 5T33MM mice.**

***A)* Full body images of mice injected with DiR-labelled HEK293-derived EVs or DiR dye control at different timepoints.** Native EVs (9 x 10^10^, as determined by NTA) or processed 5% EDS medium were labelled with DiR (5 µM). After removing the dye by ultracentrifugation, EVs were injected intravenously in depilated healthy and 5T33MM-bearing mice, 18 days after tumour inoculation. To follow up EV biodistribution over time, mice were imaged at 1 hour, 3 hours, 6 hours and 24 hours after injection using the Fluobeam 800 near-infrared fluorescence camera. Images from one mouse representative of 6 naïve and 7 5T33MM mice injected with DiR-labelled native EVs and 3 naïve and 3 5T33MM mice injected with a DiR dye are shown. Note how the DiR signal in the legs of EV-injected mice is highest after 24 hours.

***(B,C)* Comparison of the biodistribution of native EVs and DiR dye controls based on the DiR signal emitted from isolated organs.** *(B)* Representative images from one mouse per group are shown. Exposure time is either maximum exposure (1,000 ms) or the longest exposure with no saturation of the signal, as indicated. *(C)* Mean grey values quantified using ImageJ. Bars represent the mean ± SD of 1 PBS-injected naive mouse, 6 naïve and 7 5T33MM mice injected with DiR-labelled native EVs and 3 naïve and 3 5T33MM mice injected with a DiR dye control. Statistical analyses were performed using a one-tailed Mann-Whitney U-test, comparing naïve and 5T33MM mice within each condition (native EVs and DiR dye controls).


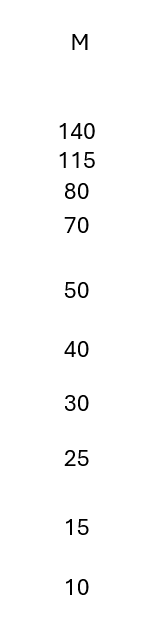

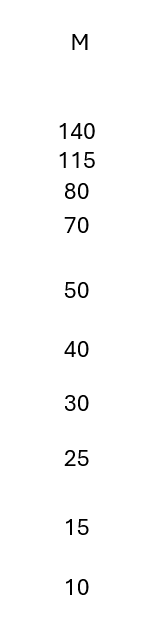

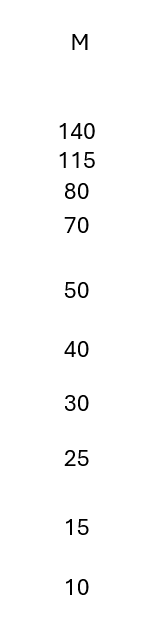

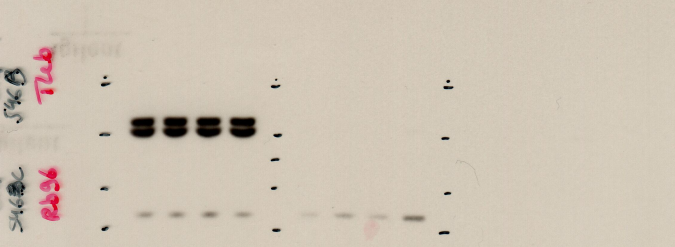
**
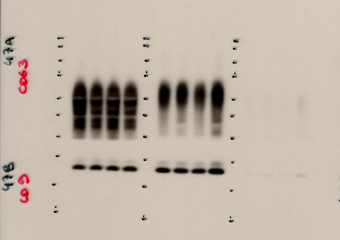
**
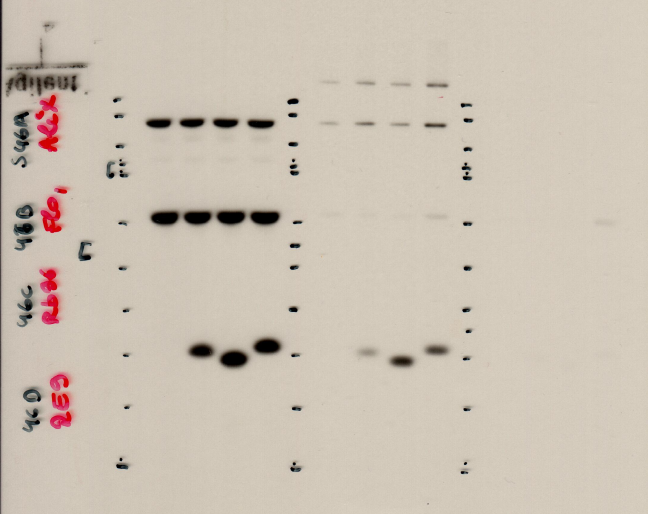

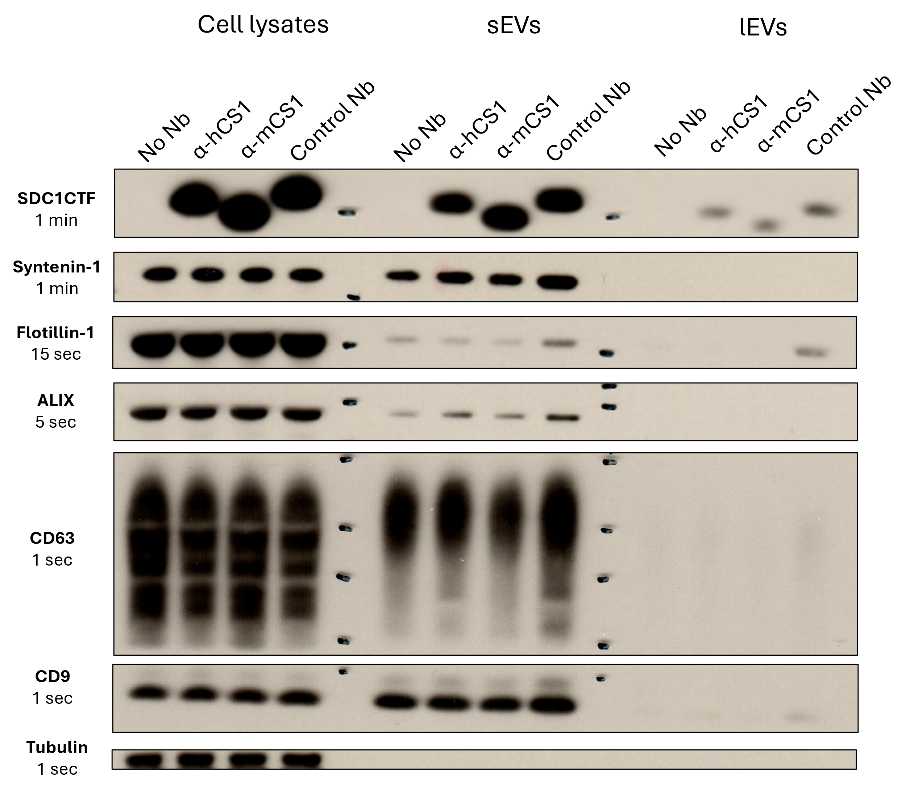


ALIX

Nb construct

Exposure time: 1s

Exposure time: 1s

Exposure time: 1s

Syntenin-1

Tubulin

CD9

CD63

Note: tubulin was developed consecutively to flotillin-1 on the same membrane, resulting in two bands.

Flotillin-1


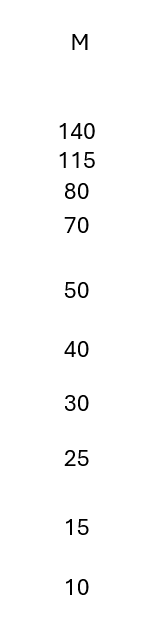

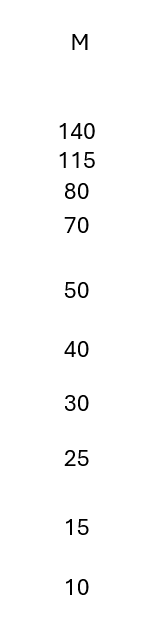

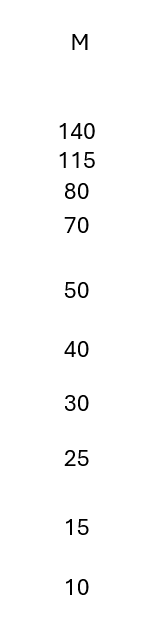

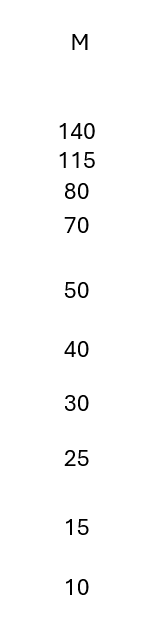

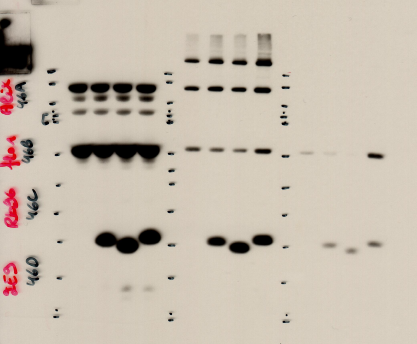
**
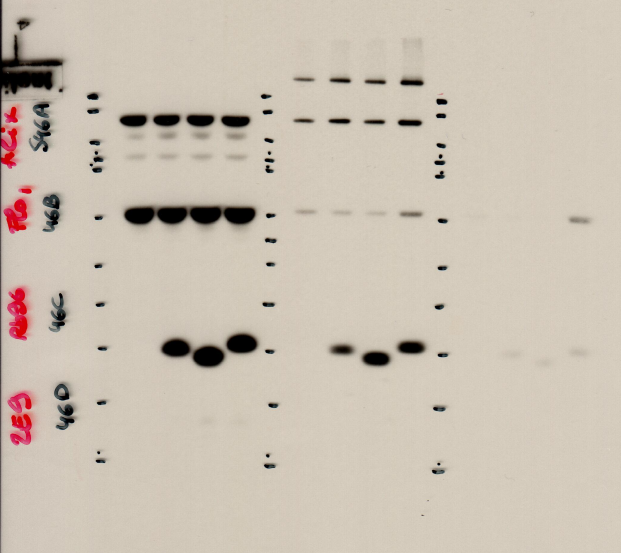
**
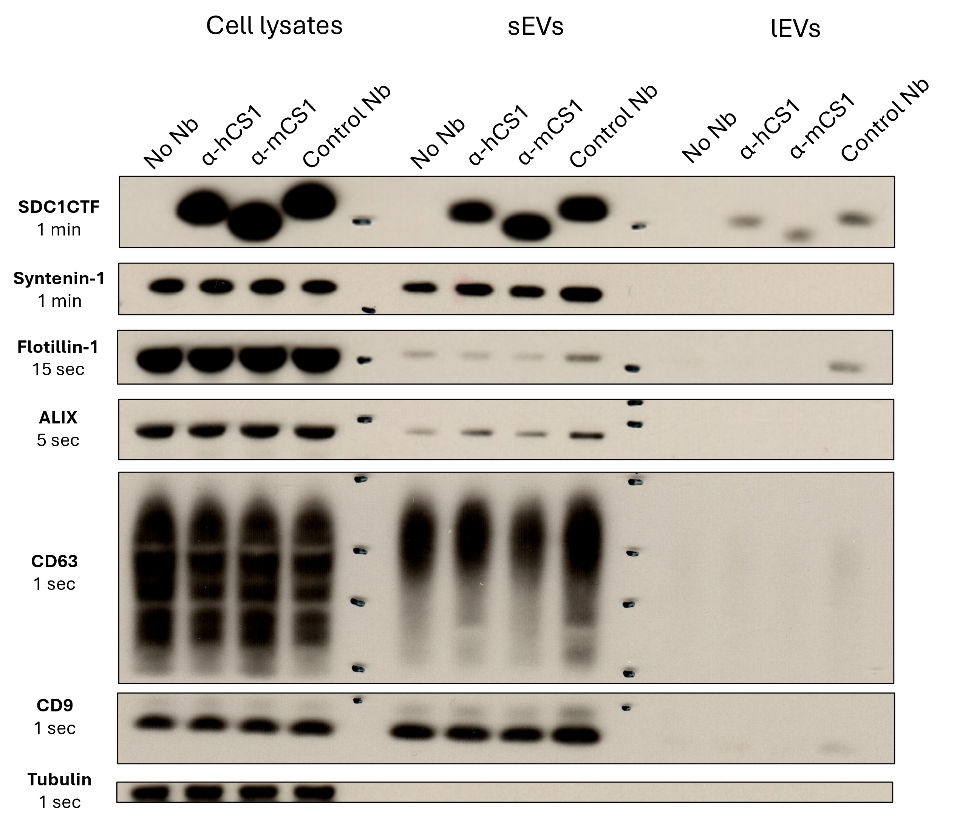
**
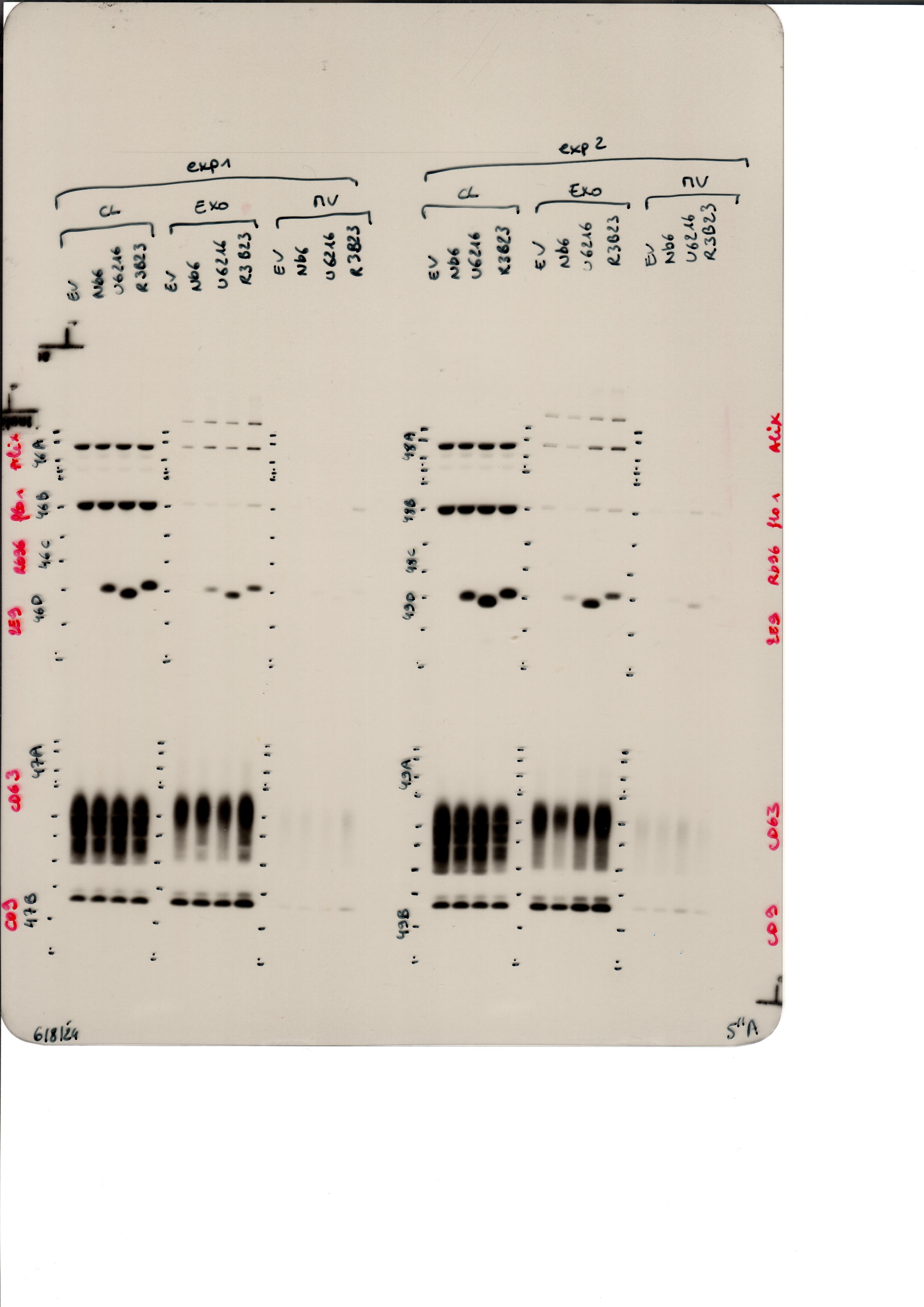
**

Nb construct

Nb construct

Nb construct

Flotillin-1

ALIX

Exposure time: 15s

Exposure time: 1 min

Flotillin-1

ALIX

Exposure time: 5s

Flotillin-1

ALIX


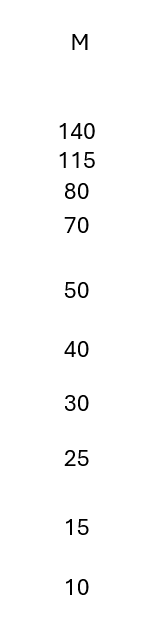

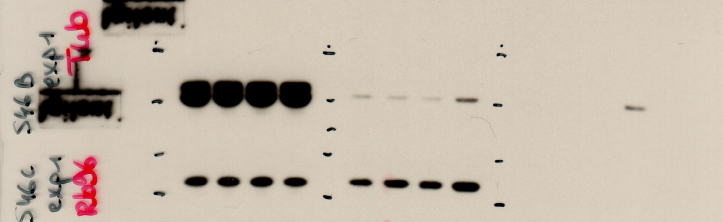

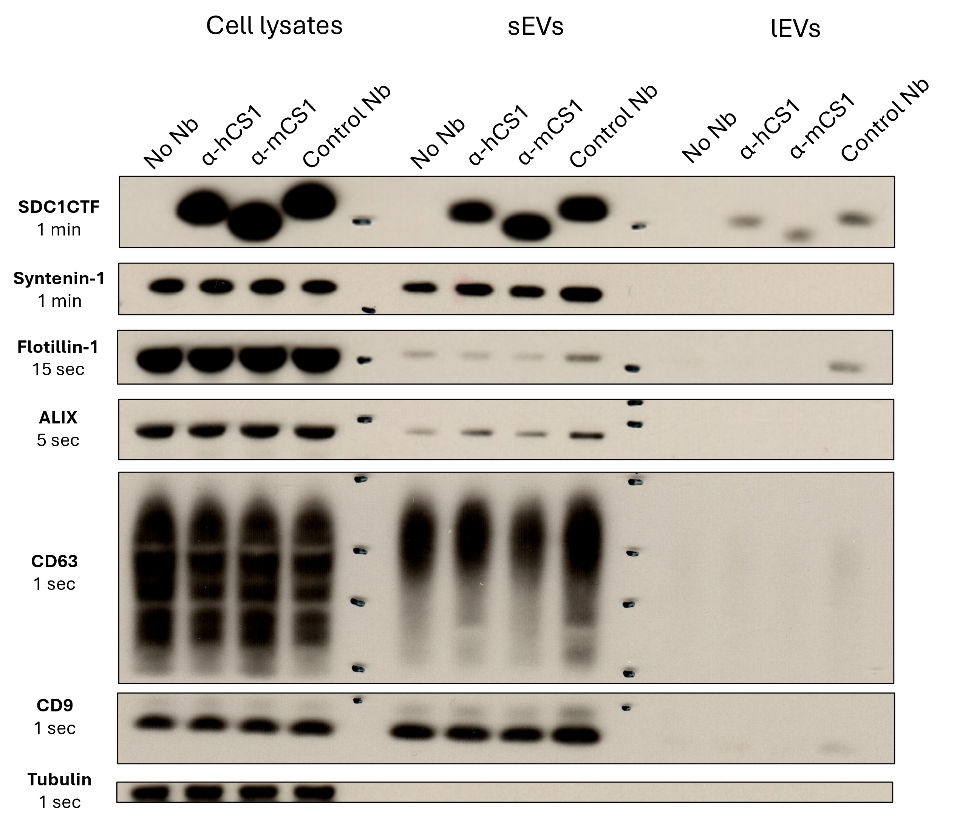


Exposure time: 1 min

Tubulin

Syntenin-1


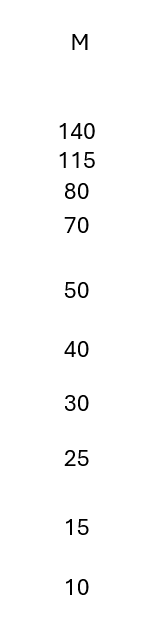


**Supplemental Figure S2: raw uncropped western blot images in support of Figure 2B.** Molecular weight ladders are indicated on the western blot images in kDa.

B.

A.

**Supplemental Figure S3: Quantifications of the optical densities for the EV markers in sEVs *(A)* and lEVs *(B)*.** lEVs were pelleted by centrifuging at 10.000 x g, after which sEVs were pelleted by ultracentrifugation at 100.000 x g. Optical density was measured for indicated proteins using ImageJ. These quantifications were made on the immunoblots shown in Supplemental Figure S2. The exposure times used for quantifications are as follows: syntenin-1 (sEVs: 1 min, lEVs: not detected), Flotillin-1 (sEVs: 1 min, lEVs: 5 min), ALIX (sEVs: 5 sec, lEVs: not detected), CD63 (sEVs: 1 sec, lEVs: 1 min), CD9 (sEVs: 1 sec, lEVs: 1 min). Bars represent mean ± SD of n = 3 for sEVs and n = 2 for lEVs. No significant differences were seen in any of the engineered sEVs compared to native sEVs (Kruskal Wallis test).


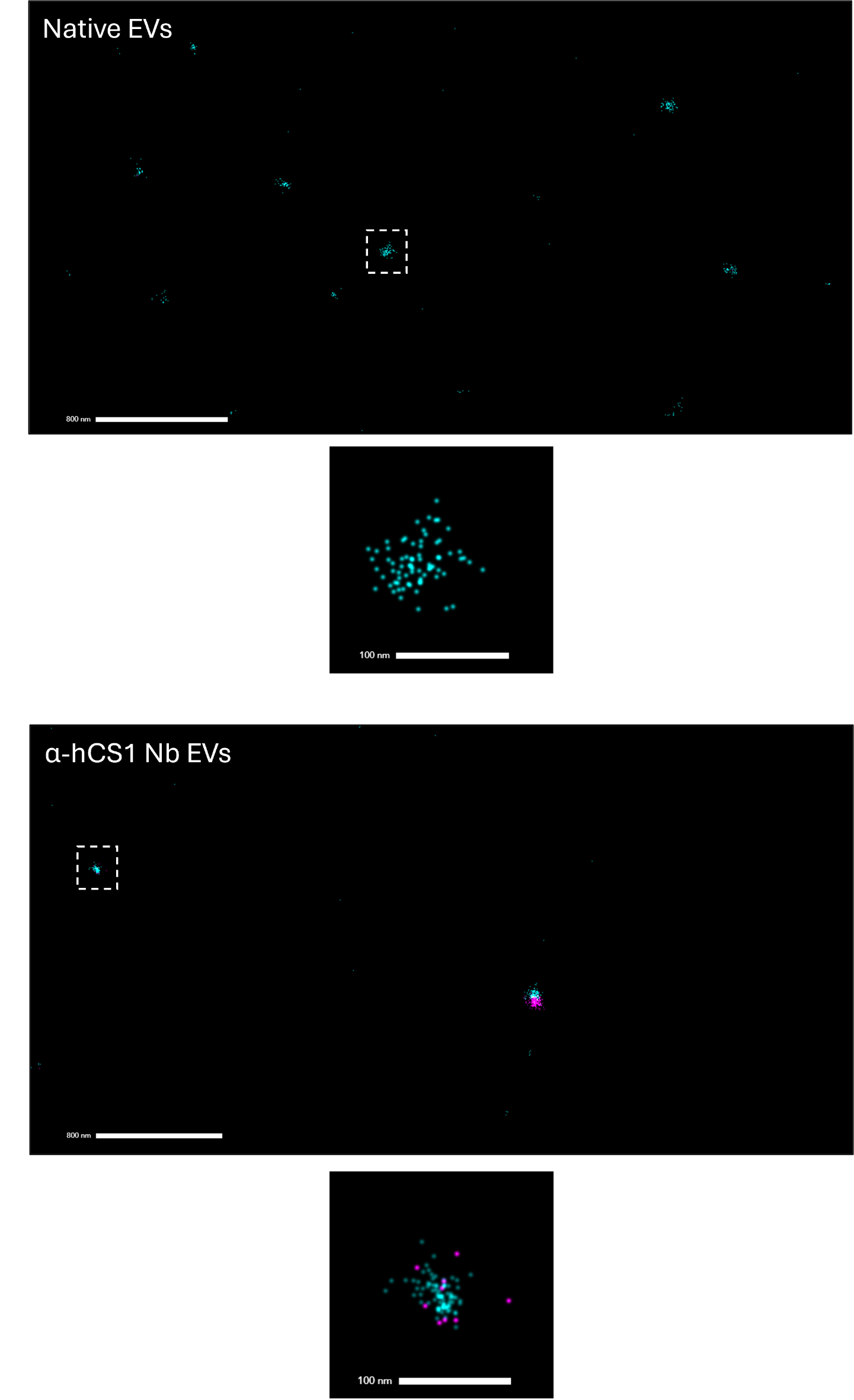


**
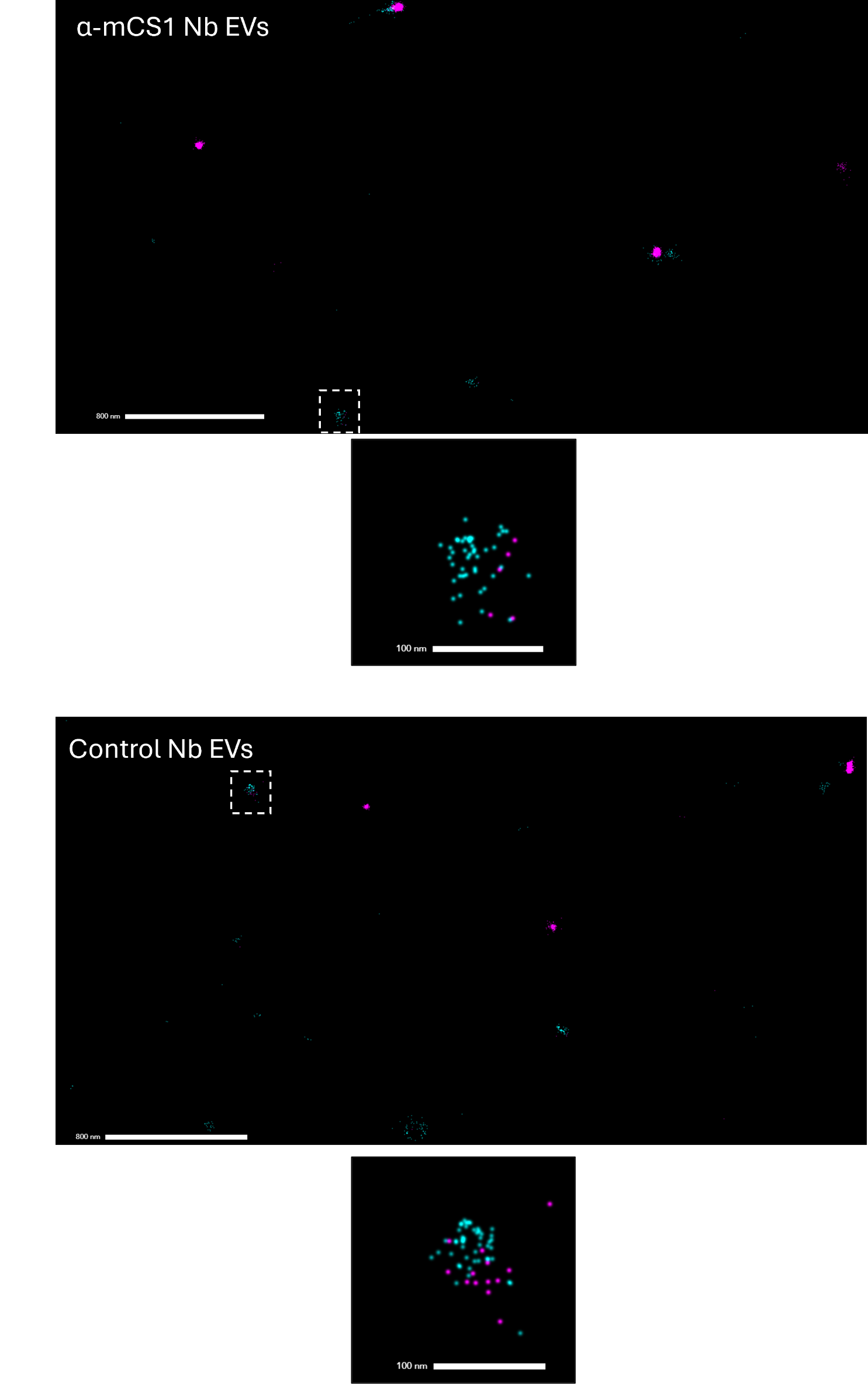
**

**Supplemental Figure S4: Tetraspanins and Nbs present on engineered HEK293-derived EVs.** Representative images for the determination of Nbs displayed on the indicated CCM EVs in Figure 2C. Blue dots indicate AF561-conjugated pan-tetraspanin antibodies (recognizing CD63, CD81 and CD9), while pink dots represent anti-V_HH_ antibodies, recognizing the Nbs. For each EV type, a general field view is given (top), with the indicated area magnified (bottom). Scale bars represent 800 nm for field views and 100 nm for magnified areas.

**
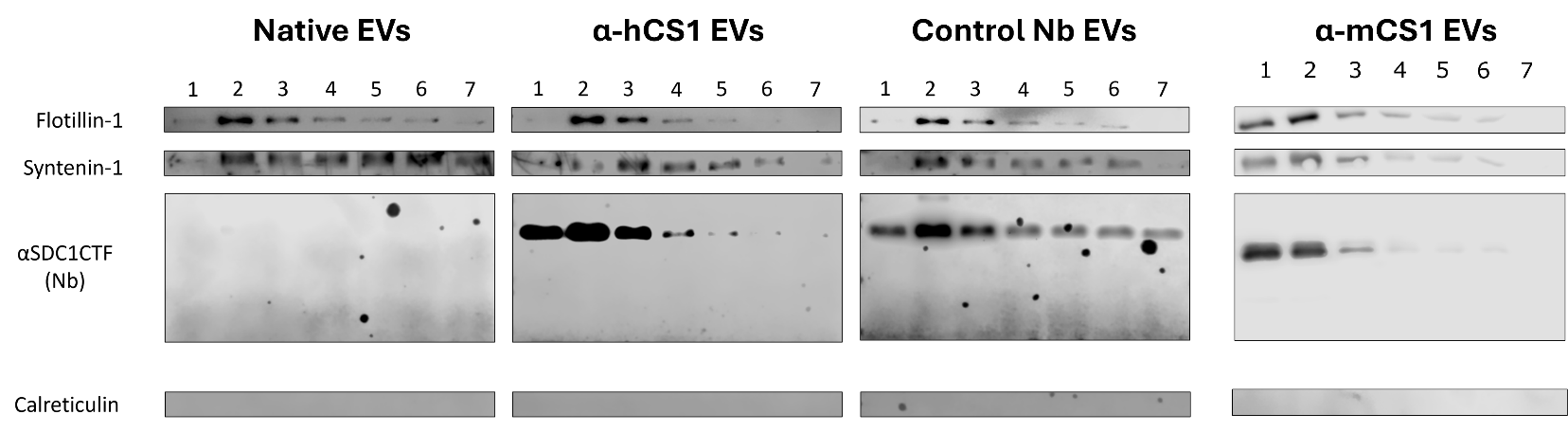
**

A.


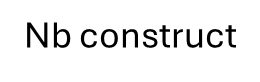

B.

**Supplemental Figure S5: Isolation of the EVs from the stably transfected HEK293 cells using size-exclusion chromatography (SEC).**

***(A)* Western blot analysis of the SEC fractions of the conditioned media from** **stably transfected HEK293 cells.** EVs were isolated by filtration and SEC from approximately 25 mL of conditioned medium derived of 25 x 10^6^ cells. SEC fractions were collected, concentrated and loaded on western blot to analyse for flotillin-1, syntenin, SDC1CTF and calreticulin. Blots from one representative experiment of 3 are shown, except for α-mCS1 Nb EVs for which n = 2.

***(B)* Quantification of the optical densities.** The optical densities (O.D.) of the bands shown in *(A)* were determined using ImageJ. Bars represent mean ± SD of 3 independent experiments for all EV types, except for α-mCS1 Nb EVs for which n = 2.


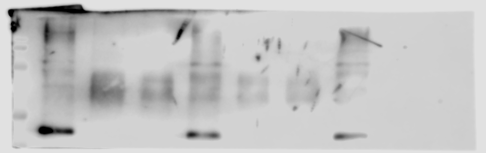
**
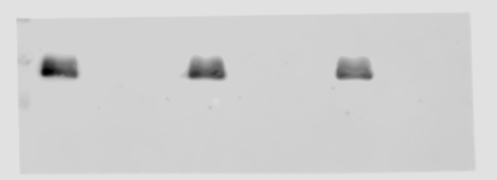
**
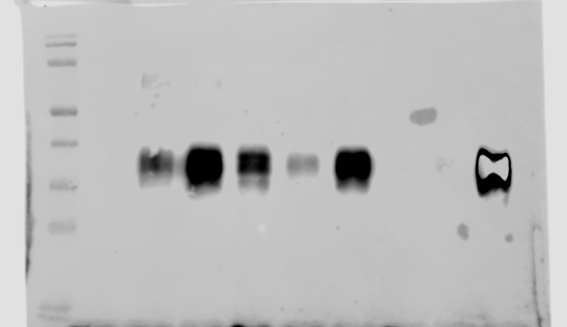

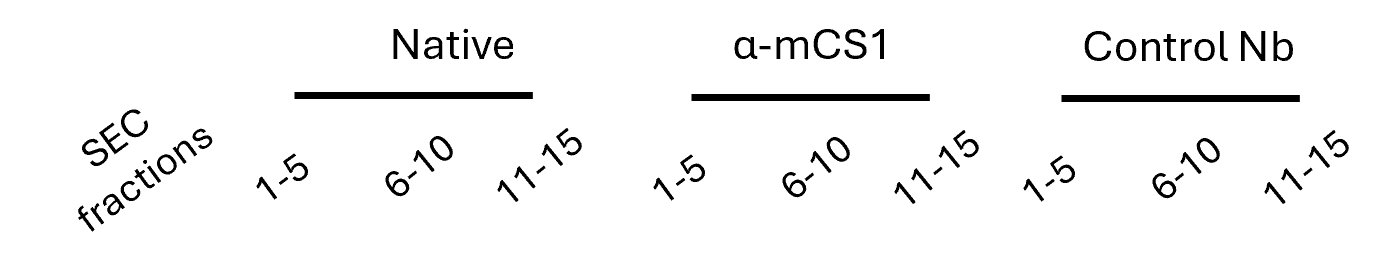

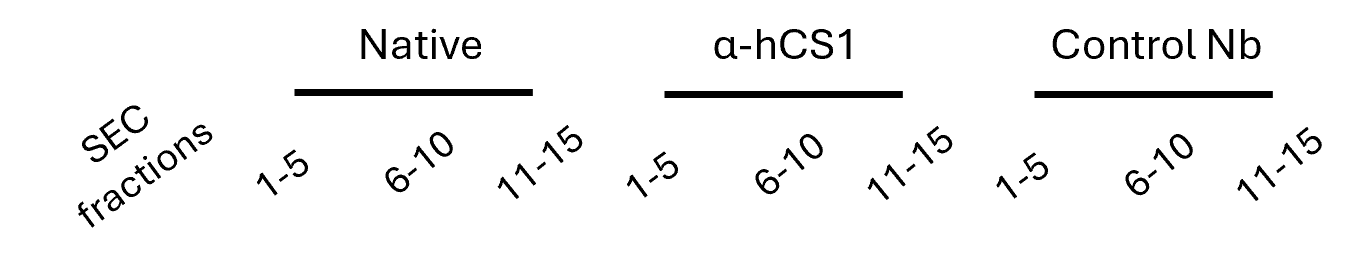
**
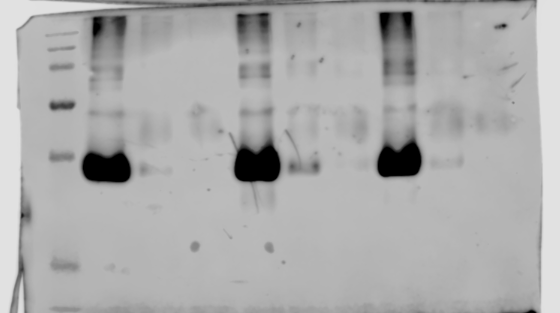

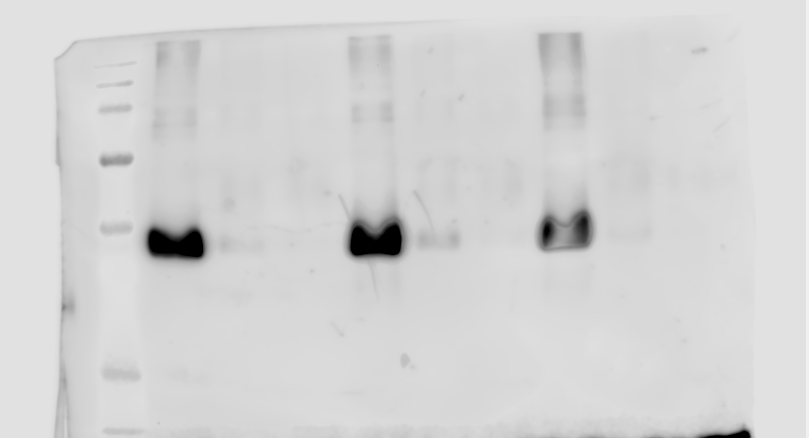

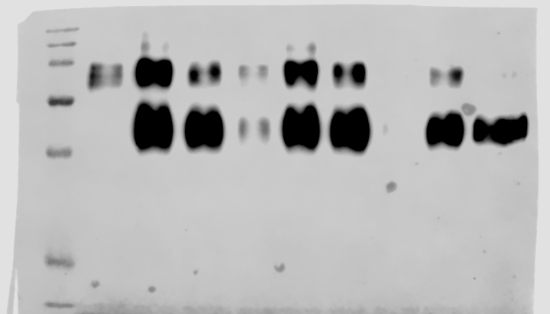
**

Uncropped Fig. 3C

Uncropped Fig. 3B

Flotillin-1

Syntenin-1

mCS1

Flotillin-1

Syntenin-1

hCS1

180

130

130

180

100

100

55

40

55

35

40

25

35

15

25

130

180

100

55

40

40

35

25

35

25

130

130

180

40

55

100

100

55

40

35

25

**Supplemental Figure S6: raw uncropped western blot images in support of Figure 3B,C.** Molecular weight ladders are indicated on the western blot images in kDa. The upper bands (70-90 kDa) likely represent CS1 dimers due to homotypic interactions^1^.

**
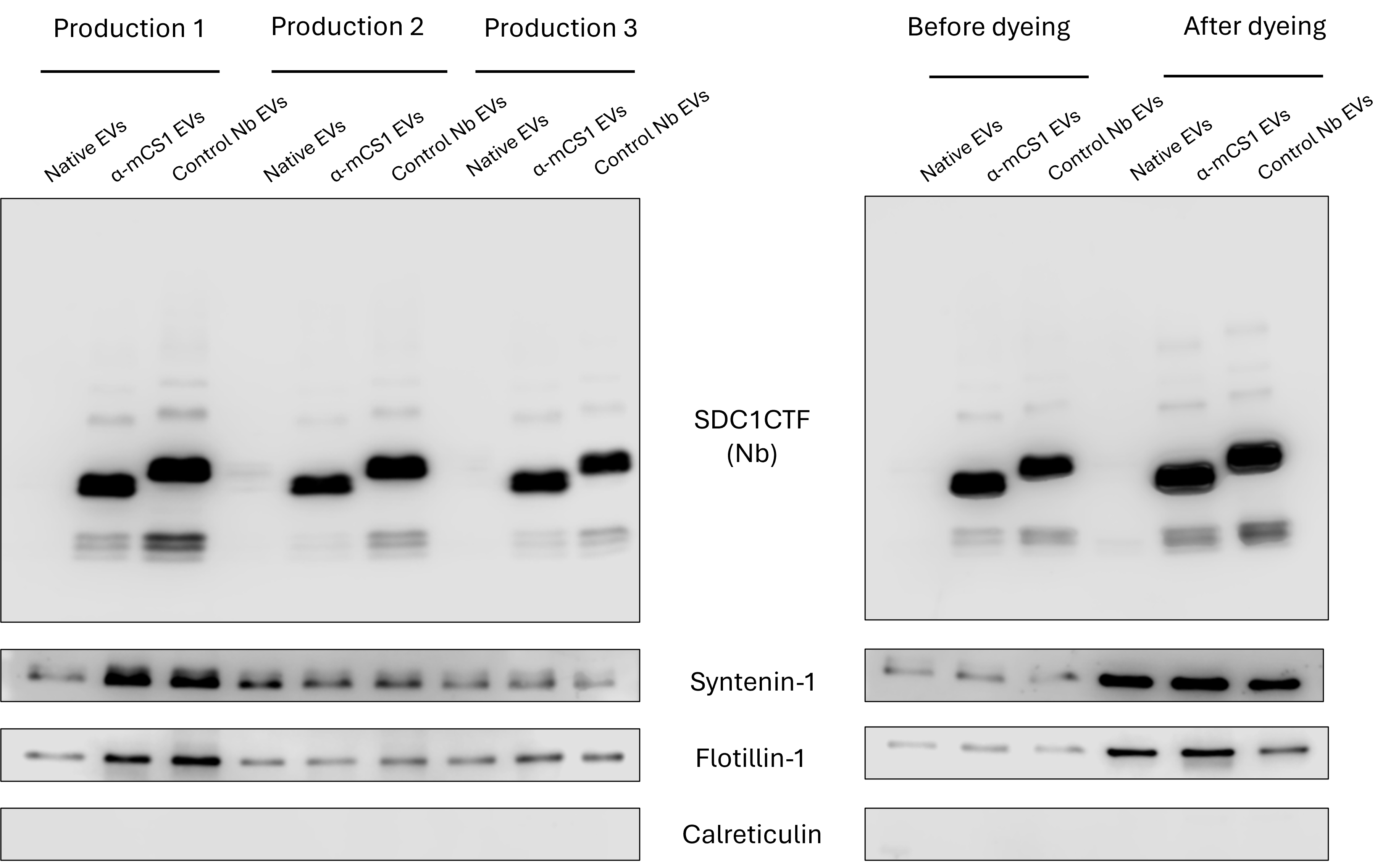
**

Nb construct

**Supplemental Figure S7:** **Quality control of the EVs after production and before injection.**

**Left: Quality controls of the individual EV productions.** Western blot analysis of EV markers (syntenin-1, flotillin-1), the EV-associated protein SDC1CTF, and the EV exclusion marker calreticulin confirming consistent EV quality across samples and experiments. For each production, five T175 flasks of each cell type were used. EVs were isolated from their conditioned medium by differential centrifugation. Three parallel batches of each EV type were produced per injection and stored at -80°C. Western blot was performed on 4% of the EVs produced for each batch.

**Right: Quality controls of pooled EVs before and after DiR dyeing.** On the day of injection, EVs were thawed, pooled from three productions, and normalized to the same particle concentration, as determined by NTA. Western blot analysis was performed on 0.1% of pooled EVs before DiR labelling and 1% after dyeing with DiR (5µM) and removing free dye by ultracentrifugation to assess conformity. The remaining EVs were injected into the 5T33MM mice.

One result representative of seven independent productions is shown.

**
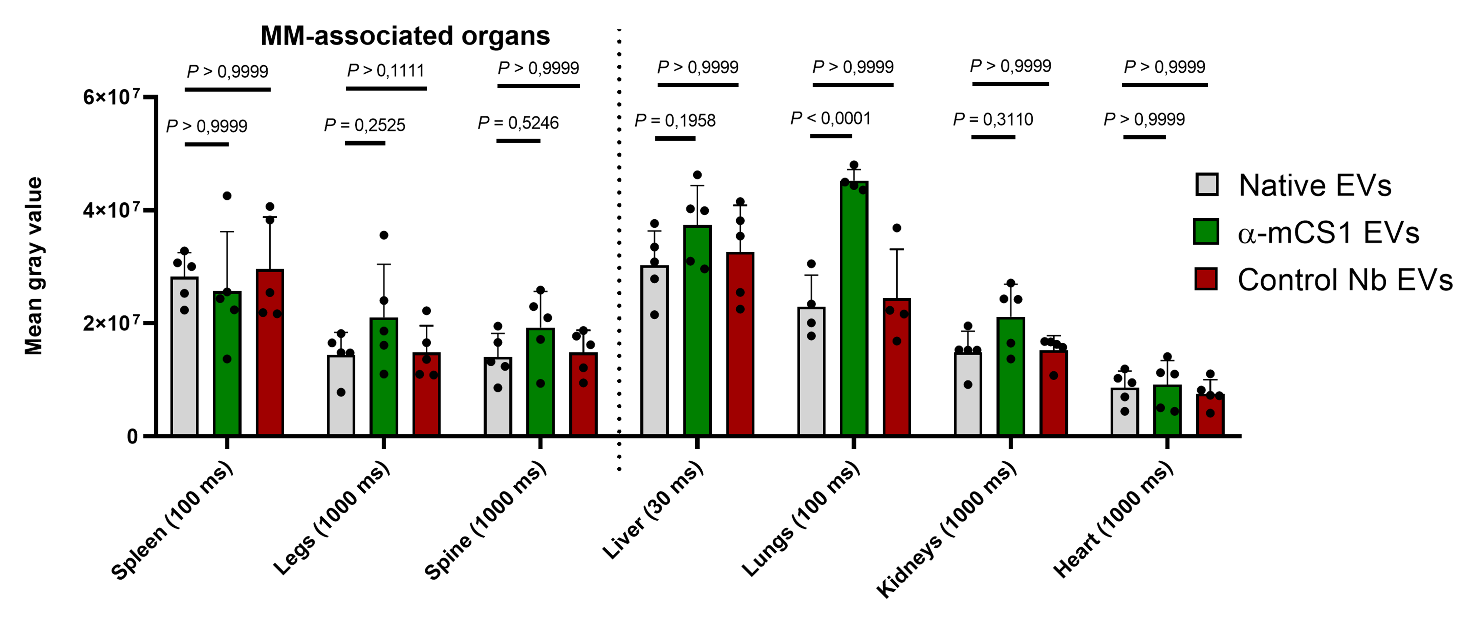
**

**Supplemental Figure S8: Biodistribution of DiR-labelled native and Nb-engineered EVs in 5T33 myeloma-bearing mice.** Native, α-mCS1 Nb and control Nb EVs (9 x 10^10^, as determined by NTA) were labelled with DiR (5 µM). After removing free dye by ultracentrifugation, EVs were injected intravenously in mice, 18 days post tumour inoculation. After 24 hours, the mice were sacrificed, and the dissected organs were imaged with a Fluobeam 800 fluorescence camera. Mean grey values of dissected organs were quantified using ImageJ. Bars represent the mean ± SD of  n = 5, except for the lungs where n = 4, as the signal of the lungs of one mouse receiving anti-CS1 Nb EVs was disturbed by clotted blood. Statistical analysis was performed using a two-way ANOVA with Bonferroni’s multiple comparison test. Statistical differences were observed only in the lungs, with no significant differences in the other organs. These data relate to the data shown in Figure 4.

**
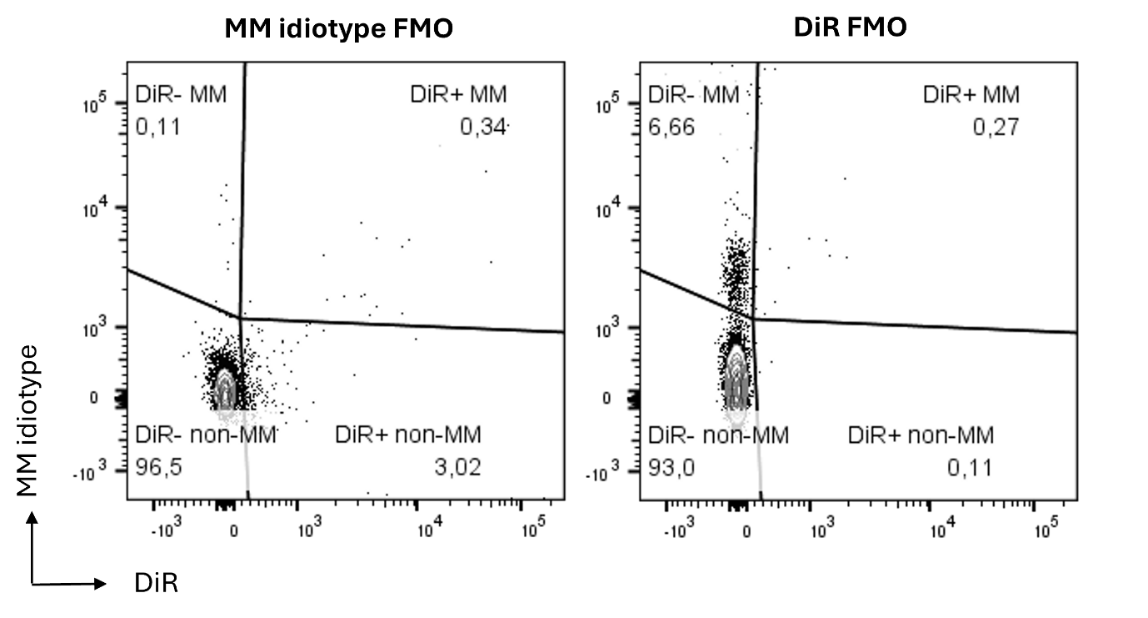
**

**MM cell marker FMO**

MM cell marker

A.

B.


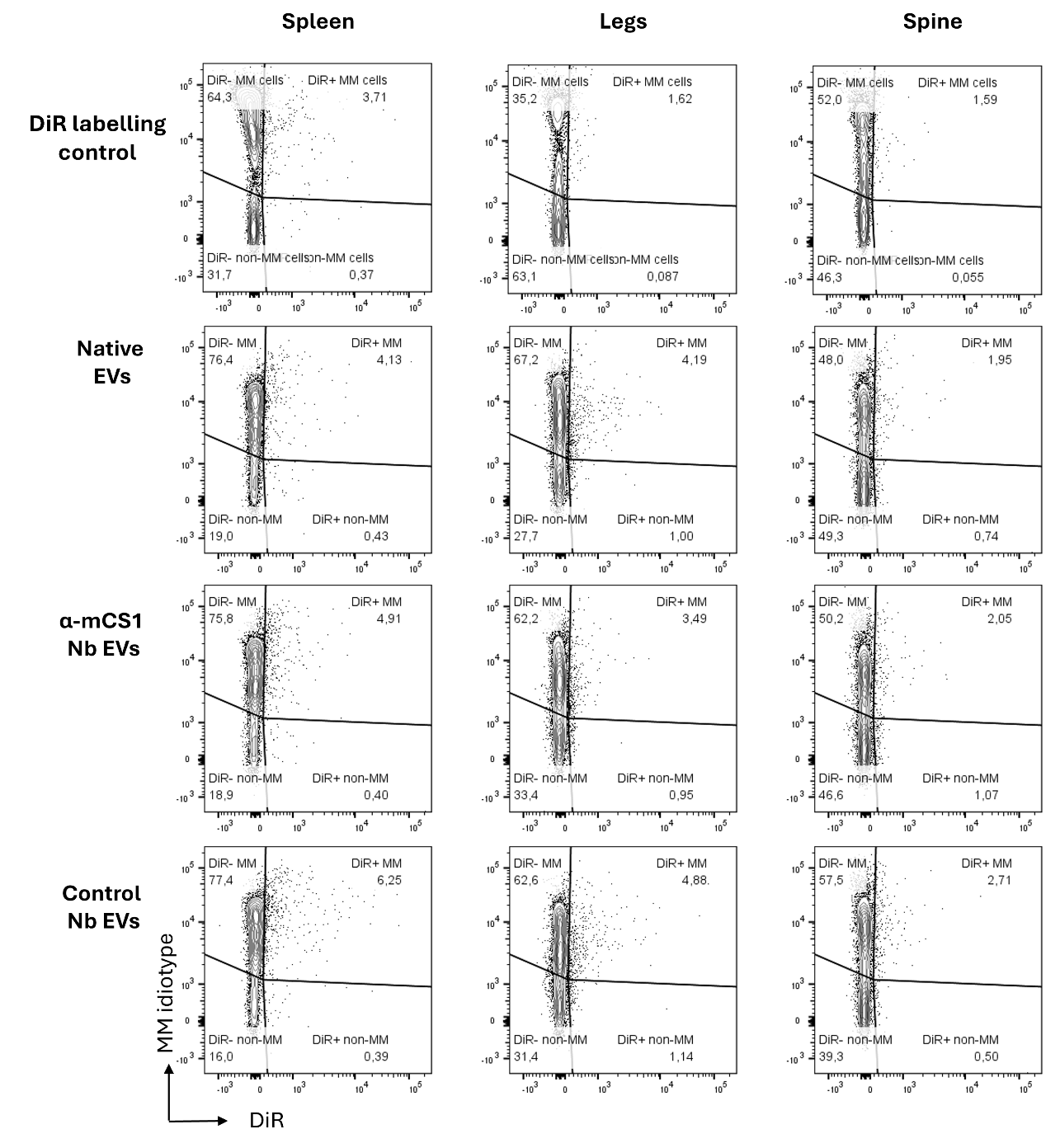


MM cell marker

**Supplemental Figure S9: Supporting information for the determination of the cellular specificity of intravenously injected native and Nb-displaying EVs towards MM and non-MM cells in 5T33MM mice.**

***(A)* Staining control used for gating MM and DiR^+^ cells.** In the MM cell marker FMO (left), the home made anti-idiotype antibody 3H2 specifically staining 5T33MM cells, was omitted from the antibody mix, while in the DiR FMO (right), mononuclear cells from a non-EV injected 5T33MM mouse was used.

***(B-E)* Cellular specificity of DiR-labelled native and Nb-engineered EVs in 5T33 myeloma-bearing mice.** *(B)* Flow cytometry data of one representative experiment, showing cells positive for the MM cell marker (5T33MM idiotype) on the y-axis and cells positive for the DiR dye on the x-axis. Gating allows determination of DiR^+^ MM and non-MM cells. *(C-E)* The % DiR^+^ cells *(C)*, the fraction of DiR^+^ MM cells *(D)* and the fraction of DiR^+^ non-MM cells *(E)*. Bars represent mean ± SD of 3 independent experiments. Statistical analysis was performed using a two-way ANOVA with Bonferroni’s multiple comparison test but revealed no statistical differences. These data relate to the data shown in Figure 5.

***(F)* Myeloma burden in the indicated organs as determined by flow cytometry.** Cells stained with the anti-idiotype 3H2 antibody were considered MM cells. These consist of both DiR^-^ and DiR^+^ MM cells gated in Supplemental Figure S9B.

**Supplemental Figure S10: Dose-escalation study of DiR-labelled α-mCS1 Nb EVs in 5T33MM diseased mice.**

***(A)* Ex vivo determined biodistribution of α-mCS1 Nb EVs based on the DiR signal emitted from isolated organs.**α-mCS1 Nb EVs were labelled with DiR (5 µM) and intravenously injected in mice 18 days post tumor inoculation at three different doses, namely full, half and quart dose corresponding to 8, 4 and 2 x 10^10^ EVs, respectively. After 24 hours, the mice were sacrificed and dissected. Dissected organs were imaged with a Fluobeam 800 fluorescence camera using indicated exposure times. Mean grey values of dissected organs were quantified using Image J.

***(B-E)* EV uptake by MM/ and non-MM cells and CS1^+^/CS1^-^ cells in the spleen, legs and spine.**Twenty-four hours post-EV injection, mononuclear cells were isolated from the spleen, legs and spine and stained with the 3H2 antibody staining the 5T33MM idiotype antigen or an anti-mCS1 antibody. DiR fluorescence was then determined in MM/non-MM (B-C) and CS1^+^/CS1^-^ (D-E) cells by flow cytometry.

***(F-G)* Myeloma burden in the indicated organs as determined by flow cytometry**. To determine tumor burden, mononuclear cells were stained either with the anti-idiotype 3H2 antibody (F) or an anti-mCS1 antibody (G). Bars represent the mean ± SD of n = 3.

Statistical analysis was performed with a one-way ANOVA with Tukey’s multiple comparison test.

**
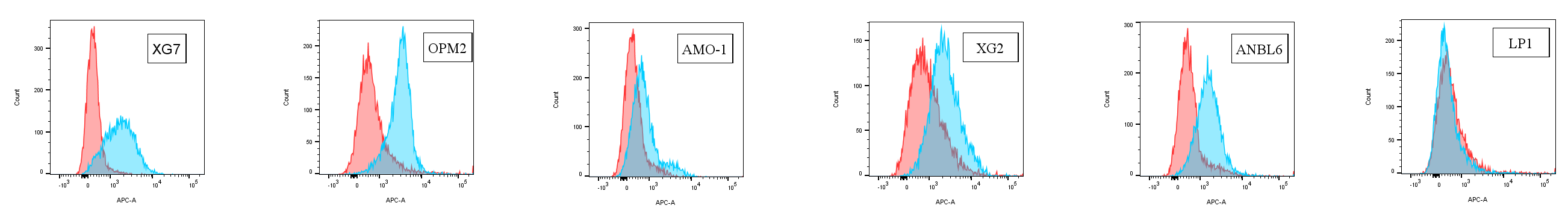
**

B.

A.

**Supplemental Figure S11:** **CS1 surface expression on the OPM-2 human myeloma cell line.**

***(A)* Histogram for CS1 surface staining and *(B*) the MFI for three independent experiments.** OPM-2 cells were stained with an α-hCS1 antibody or isotype control antibody. Bars represent mean ± SD of 3 independent experiments. Statistical analysis was done using a one-tailed Mann-Whitney U-test.

**Supplemental table S1:** Properties of α-hCS1, α-mCS1 and control Nbs.

| **Target** | **Kd (nM)** | **Sequence** | **Ref** |
| --- | --- | --- | --- |
| **hCS1** | 72 | DVQLVESGGGLVQAGGSLRLSCAASGRTFEDYFMA  WFRQIPGKEREFVAAVGWNTGRGYYTDSVKGRFTIS  RDSAKNTLYLQMDSLKPEDTAVYYCNAPLQSLDRRL  PGPYWGQGTQVTVSS | ^2^ |
| **mCS1** | 154 | QVQLQESGGGLVQPGGSLRLSCAASGFTFSSTAMS  WARQAPGKGLEWVSSIYSDGSTSYADSMKGRSTISR  DNAKNTVYLQMNSLKPEDTAVYYCAIGDTGWGRVG  QGTQVTVSS | ^3^ |
| **Control Nb binding the 5T2MM model idiotype** | 7,8 | QVQLQESGGGSVQAGGSLRLSCAASGDTGYMGWF  RQAPGKEREGVAVINSDSGVGSTYYADSVKGRFTISR  DNAKNTVYLQMNSLKPEDTAIYYCAAGHFSDYVSPW  TWREIYRYNVWGQGTQVTVSS | ^4^ |

**Supplemental table S2:** Primary and secondary antibodies used for western blot analyses.

| **Primary antibodies** | | | |
| --- | --- | --- | --- |
| **Target** | **Host** | **Clone/Catalog no.** | **Company** |
| **hCS1** | Rabbit | E5C4M/#98611 | Cell Signaling Technology (CST) |
| **mCS1** | Rat | MAB4628 | R&D Systems |
| **Syntenin-1** | Rabbit | #19906 | Abcam |
| **Flotillin-1** | Rabbit | D2V7J/#18634 | CST |
| **Calreticulin** | Rabbit | #2891 | CST |
| **Tubulin** | Rabbit | #2144 | CST |
| **SDC1CTF** | Mouse | 2E9 | In house^5^ |
| **Secondary antibodies (HRP-linked)** | | | |
| **Target** | **Host** | **Clone/Catalog no.** | **Company** |
| Mouse IgG | Horse | #7076 | CST |
| Rabbit IgG | Goat | #7074 | CST |
| Rat IgG | Goat | NA935 | Cytiva |

**Supplemental references:**

1. Cannons, J. L., Tangye, S. G. & Schwartzberg, P. L. SLAM Family Receptors and SAP Adaptors in Immunity. *Annual Review of Immunology* **29**, 665–705 (2011).

2. Hanssens, H. *et al.* Scrutiny of chimeric antigen receptor activation by the extracellular domain: experience with single domain antibodies targeting multiple myeloma cells highlights the need for case-by-case optimization. *Front. Immunol.* **15**, (2024).

3. De Veirman, K. *et al.* CS1-specific single-domain antibodies labeled with Actinium-225 prolong survival and increase CD8+ T cells and PD-L1 expression in Multiple Myeloma. *OncoImmunology* **10**, 2000699 (2021).

4. Lemaire, M. *et al.* Imaging and radioimmunotherapy of multiple myeloma with anti-idiotypic Nanobodies. *Leukemia* **28**, 444–447 (2014).

5. Schulz, J. G. *et al.* Syndecan 3 Intramembrane Proteolysis Is Presenilin/γ-Secretase-dependent and Modulates Cytosolic Signaling *. *Journal of Biological Chemistry* **278**, 48651–48657 (2003).
